# Dynamic Electrode-to-Image (DETI) mapping reveals the human brain’s spatiotemporal code of visual information

**DOI:** 10.1371/journal.pcbi.1009456

**Published:** 2021-09-27

**Authors:** Bruce C. Hansen, Michelle R. Greene, David J. Field

**Affiliations:** 1 Colgate University, Department of Psychological & Brain Sciences, Neuroscience Program, Hamilton New York, United States of America; 2 Bates College, Neuroscience Program, Lewiston, Maine, United States of America; 3 Cornell University, Department of Psychology, Ithaca, New York, United States of America; University of Glasgow, UNITED KINGDOM

## Abstract

A number of neuroimaging techniques have been employed to understand how visual information is transformed along the visual pathway. Although each technique has spatial and temporal limitations, they can each provide important insights into the visual code. While the BOLD signal of fMRI can be quite informative, the visual code is not static and this can be obscured by fMRI’s poor temporal resolution. In this study, we leveraged the high temporal resolution of EEG to develop an encoding technique based on the distribution of responses generated by a population of real-world scenes. This approach maps neural signals to each pixel within a given image and reveals location-specific transformations of the visual code, providing a spatiotemporal signature for the image at each electrode. Our analyses of the mapping results revealed that scenes undergo a series of nonuniform transformations that prioritize different spatial frequencies at different regions of scenes over time. This mapping technique offers a potential avenue for future studies to explore how dynamic feedforward and recurrent processes inform and refine high-level representations of our visual world.

## Author summary

The visual information that we sample from our environment undergoes a series of neural modifications, with each modification state (or visual code) consisting of a unique distribution of responses across neurons along the visual pathway. However, current noninvasive neuroimaging techniques provide an account of that code that is coarse with respect to time or space. Here, we present dynamic electrode-to-image (DETI) mapping, an analysis technique that capitalizes on the high temporal resolution of EEG to map neural signals to each pixel within a given image to reveal location-specific modifications of the visual code. The DETI technique reveals maps of features that are associated with the neural signal at each pixel and at each time point. DETI mapping shows that real-world scenes undergo a series of nonuniform modifications over both space and time. Specifically, we find that the visual code varies in a location-specific manner, likely reflecting that neural processing prioritizes different features at different image locations over time. DETI mapping therefore offers a potential avenue for future studies to explore how each modification state informs and refines the conceptual meaning of our visual world.

This is a *PLOS Computational Biology* Methods paper.

## Introduction

Upon viewing a new scene, the brain transforms the ambient light array hitting the retinae into semantically meaningful content that enables intelligent behavior, all within the first 300 ms of viewing. However, the series of representational transformations that support visual analysis of scenes are not well-understood. At the most fundamental level, we know that visual information is processed differentially by multiple neural populations that act like nonlinear filters, each coding for specific types of information in narrow bands of spatial frequency and orientation [1–3]. Further, because real-world environments are broadband in both spatial frequency and orientation, each location within a given scene will simultaneously activate a range of tuned visual neurons [4–10]. Given this, recent efforts have used visual filter-based encoder models to predict fMRI-defined patterns of blood oxygen-level dependent (BOLD) activity based on real-world scene inputs [11–13]. Such voxel-wise encoder models of BOLD signals have provided insight into the nature of how humans internalize external scene information, and on how the neural code in early visual cortices maps onto and supports higher level semantic representations [14–17]. However, those results are based on a static view of the early visual code (due to the temporal limitations of fMRI). The neural code for visual information is highly dynamic as local and long-range recurrent processes act to change the local activity patterns that are evoked by scenes over a short period of time [18–20]. However, those dynamic transformations are not yet well-characterized, thus hindering our understanding of how they enable the construction of a meaningful representation of our visual world.

Electroencephalography (EEG) has excellent temporal resolution and has been used to characterize the time-varying nature of visual information in real-world scenes [21–24]. Nonetheless, EEG suffers from scalp interference and dipole cancellation on the scalp, so those efforts have only provided a very coarse estimate of those neural dynamics, with very little insight into how local scene information is encoded in the early visual cortices and transformed over time. This is unfortunate because the early spatiotemporal transformations of visual information likely shape representations in higher-level cortical networks, ultimately shaping scene-related semantic processes [14,21,25–28]. For instance, multiple networks across lateral occipitotemporal, dorsal, ventral temporal, and medial temporal cortices have all been shown to possess a retinotopic organization and relative selectivity to different spatial frequencies [29–35].

**Fig 1:**
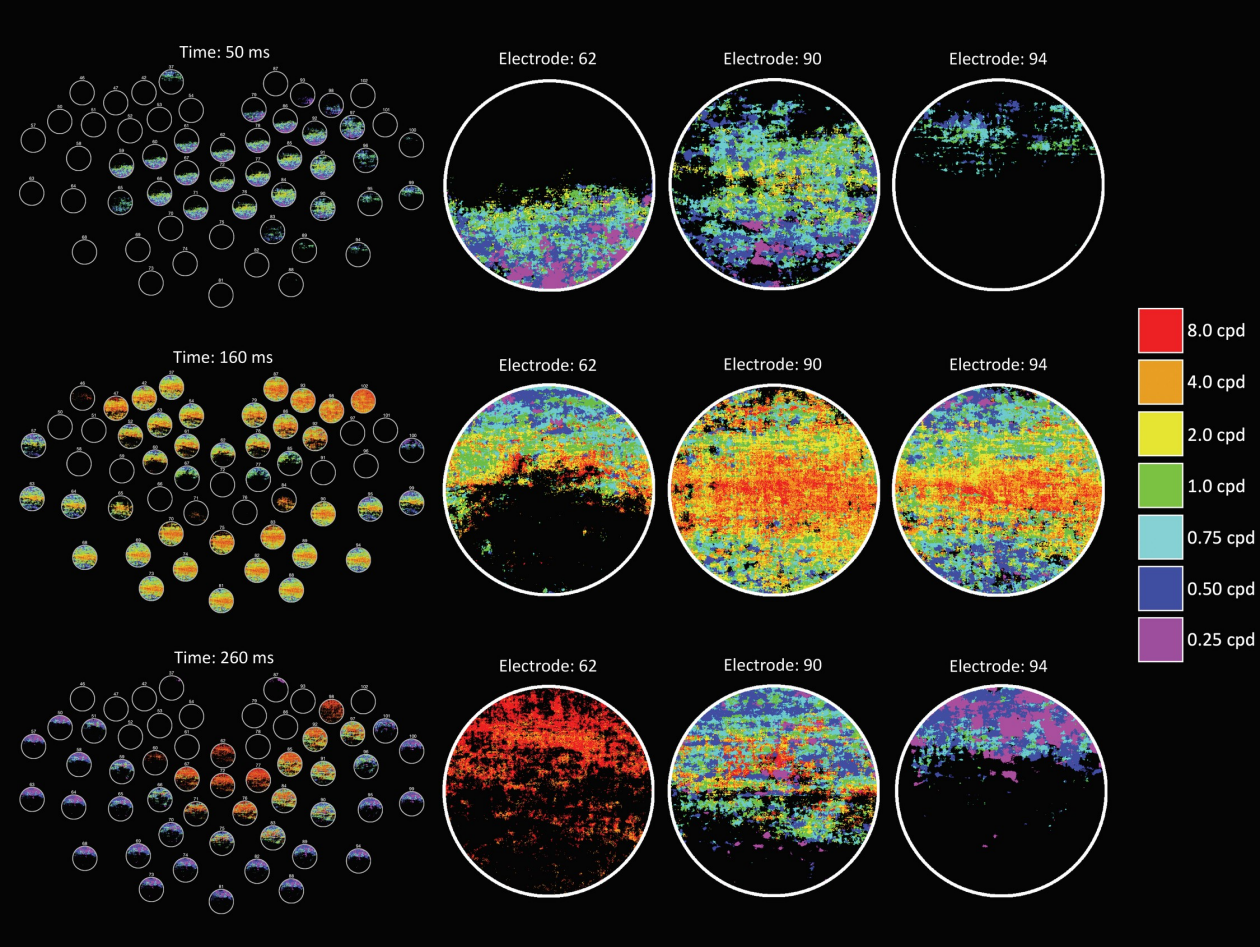
Example DETI maps from the image-general analysis at different time points. The movie version of this figure can be downloaded here https://pbsc.colgate.edu/~bchansen/HansenGreeneField2021/HansenGreeneField_Figure1_Movie.mp4. The left-hand column shows a topographical map of the posterior electrodes, illustrating the variation of DETI maps across that scalp region. On the right-hand side, each column shows the spatiotemporal evolution of the visual code for different electrodes (each row corresponds to the time given on the left-hand side). The color bar shows the spatial frequency tuning peak (in cycles per degree; cpd) of the encoder that was mapped to each pixel in the DETI maps. Note that the maps are circular because the stimuli were windowed with a circular window (see Materials & Methods).

**Fig 2:**
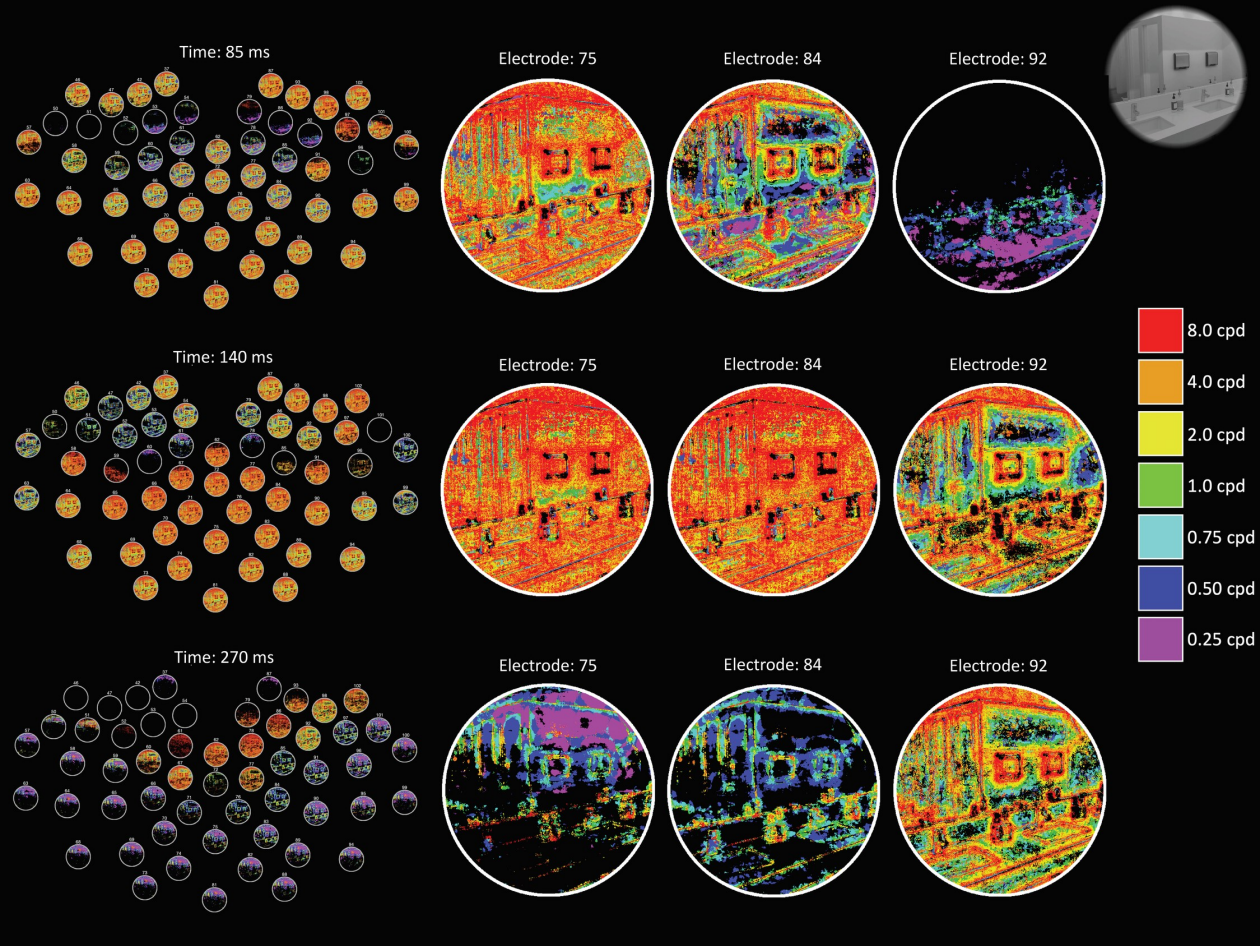
Example DETI maps from the image-specific analysis at different time points. The movie version of this figure can be downloaded here https://pbsc.colgate.edu/~bchansen/HansenGreeneField2021/HansenGreeneField_Figure2_Movie.mp4. The layout and details of this figure are identical to **Fig 1**, except here we are showing the spatiotemporal evolution of the visual code for an example image (shown in the upper right-hand corner).

This study introduces dynamic electrode-to-image (DETI) mapping: an analytical approach to map time-varying neural signals from visual evoked potentials (VEPs) to every pixel location of complex real-world scenes. This technique offers the ability to visualize and analyze the spatiotemporal evolution of visual encoding across the early stages of visual processing. To circumvent the problems inherent in EEG measures, we mapped the localized outputs of a spatial frequency tuned log-Gabor encoding model to different VEPs within a geometric state-space framework. Specifically, we measured the correspondence between the high-dimensional output variation produced by our encoding model at every location within large-field visual scenes and the response variation of VEPs measured at each electrode across the posterior region of the scalp. At its heart, our method reduces the dimensionality of VEP signals measured at each electrode at different points in time and then maps those signals via an encoding model to each pixel within and across a relatively large set of images. This geometric response space mapping procedure enables the mapping to take place across large sets of scenes (**Fig 1**) as well as for individual scenes (**Fig 2**). Specifically, this method provides 1) a general spatiotemporal view of scene encoding over an entire set of images, thereby allowing visualization of the general coding strategy over time, and 2) a scene-specific spatiotemporal view to visualize the various transformations that each scene undergoes over time. Further, this technique offers a rich source of spatiotemporal data to explore a wide variety of questions concerning the various transformational states of visual coding once thought impossible to address with EEG measures.

In this report, we took advantage of the local coding abilities of this approach to characterize the early visual filter-based transformational states of scenes. That characterization revealed that scenes undergo a series of nonuniform transformations that prioritize different spatial frequencies at different regions of scenes. Further, the spatiotemporal visual code varies in a location-specific manner, likely reflecting the underlying principles of the early visual code, thereby offering a potential avenue for future studies to explore how each transformational state informs and refines the conceptual meaning of our visual world. Finally, it is important to note that although the results we show here point to an interesting spatiotemporal response across different electrodes, we are not arguing that this is all that is represented in the electrodes’ activity. The electrodes may well respond differentially to higher level features of the scenes. However, we believe the spatiotemporal responses described in this study are an important first step.

## Results

**Fig 3:**
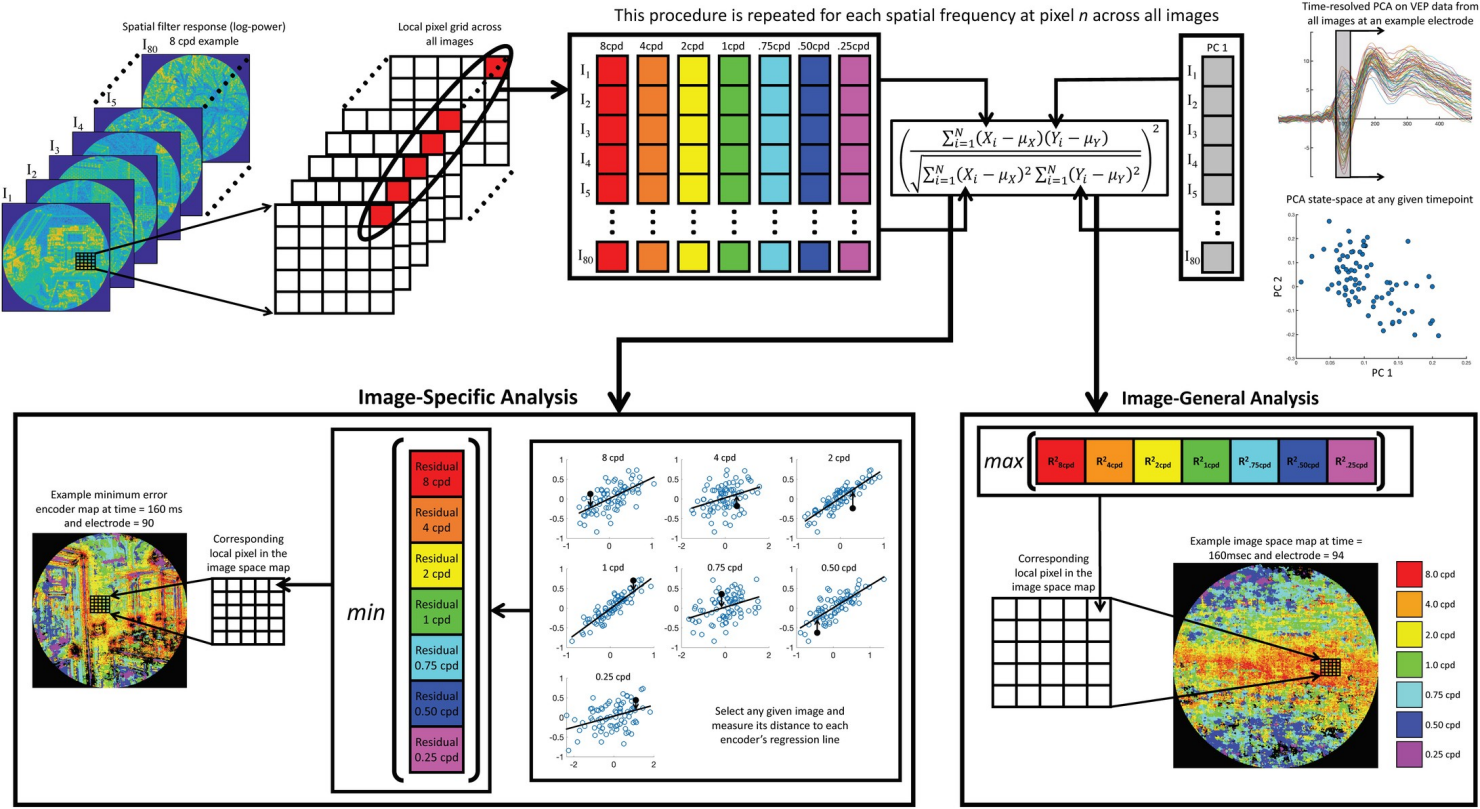
Schematic illustration of the DETI mapping procedure. All stimuli were represented in a log-Gabor filter power response space defined by seven peak SFs at each pixel coordinate across all stimuli (8 cpd encoder response examples in the upper left). For any given time point and electrode, and at any given pixel coordinate, we sampled the filter responses across all images for a given encoder and assembled them into an 80 x 1 array (this was repeated for each encoder). Then, for any given time point and electrode, each encoder’s 80 x 1 filter response array was regressed against the first PC taken from VEP data evoked by all images at the corresponding time point. Two types of maps were then produced: image-general maps (bottom right) and image-specific maps (bottom left). The image-general tagged each pixel coordinate with the SF of the encoder that had the highest R^2^. Similarly, the image-specific analysis was designed to find the encoder that could best predict the VEP variability across all images captured by the first PC for any given stimulus. However, instead of using the best fit across all images, this analysis finds the encoder regression fit that had the shortest distance (i.e., smallest residual) to a given image and tagged the corresponding pixel location with that encoder’s SF.

The dynamic electrode-to-image (DETI) mapping procedure can be broken down into three pipeline operations followed by two different mapping procedures (illustrated in **Fig 3**). The first pipeline operation projects high-dimensional VEP data into a lower dimensional space via time-resolved principal component analysis (PCA). The second operation uses an encoding approach to represent each pixel of each image with one of seven different spatial frequency peaks. The third and final pipeline operation links the lower-dimensional VEP data to each pixel in the encoder space. From there, DETI maps can be constructed for each electrode based on all scene stimuli (an image-general analysis) or for each scene stimulus (image-specific analysis). The following subsections are organized as follows: Section 1) Outlines each DETI pipeline operation and subsequent mapping procedures; Section 2) Will validate the primary metric used to generate DETI maps; and Section 3) Will present the DETI mapping results at two levels of analysis–i.e., Section 3.1: image-general and Section 3.2: image-specific), as well as the results of our analyses on the DETI maps for each level of analysis within Sections 3.1 and 3.2.

### 1.1 Time-resolved dimension reduction of VEP data

The EEG data that were used to construct visual evoked potentials (VEPs) were recorded from human participants (n = 24) while they viewed 80 scene images sampled from a variety of environments (see the Materials & Methods section for further detail). We focused our analyses on the posterior scalp electrodes (54 in total; see **S1A Fig**) because VEPs recorded at those sites carry retinotopically selective spatial frequency (SF) information [36]. To reduce the high-dimensionality of each participant’s VEP dataset (54 electrodes X 500 time points), we applied time-resolved PCA at each electrode across all scene evoked VEPs (variables) and time points (observations) in steps of 5 ms (for computational efficiency), centered within a 41 ms temporal window (±20 ms from a given time point), with each time point in the temporal window serving as feature. We chose a 41 ms temporal window as it is sufficiently broad to capture the full width at half magnitude of most VEP deflections. We tested narrower (21 ms) and broader (61 ms) window sizes and found that they only had modest effects on the mapping results. Across all participants and time points, the first two PCs were found to explain 93.2% (median) of the VEP variance, with PC 1 accounting for 76.8% of the variance. The first PC’s eigenvector for each electrode and window-centered time point was therefore used to define a uni-dimensional ‘space’ that could be mapped to each scene location within a space defined by our log-Gabor encoder model. That is, the goal of the DETI mapping procedure is to find a representational space that maps neural responses to images. By reducing the dimensionality of the VEP responses using PCA, we can represent each scene as a data point in a neural response space. From there, we can assess the extent to which that space can be explained with an image representation, in this case, a log-Gabor filter energy encoder.

### 1.2 Log-Gabor encoder model

We used log-Gabor filter response power to model the response variability of differently tuned neurons at each location in our stimuli [37–38] (see the Materials & Methods section for more detail). Briefly, the model consisted of seven filters, each tuned to a different peak SF (0.25, 0.50, 0.75, 1, 2, 4, or 8 cycles per degree; cpd) and all orientations (i.e., a log ‘doughnut’ filter in the Fourier domain)–refer to **Fig 3** for examples. The code to build the encoders and generate the encoder space can be downloaded here https://pbsc.colgate.edu/~bchansen/HansenGreeneField2021/EncoderModel.zip. While the focus of this study was on SF, we nevertheless built a set of filter encoders that spanned all SFs centered on eight different orientations (0–157.5° in steps of 22.5°) and report some of those mapping results as Supplementary Material. We emphasize that this procedure can generalize to any parameterized encoding model.

### 1.3 Linking VEP Data to encoder space and DETI mapping procedures

**Fig 4:**
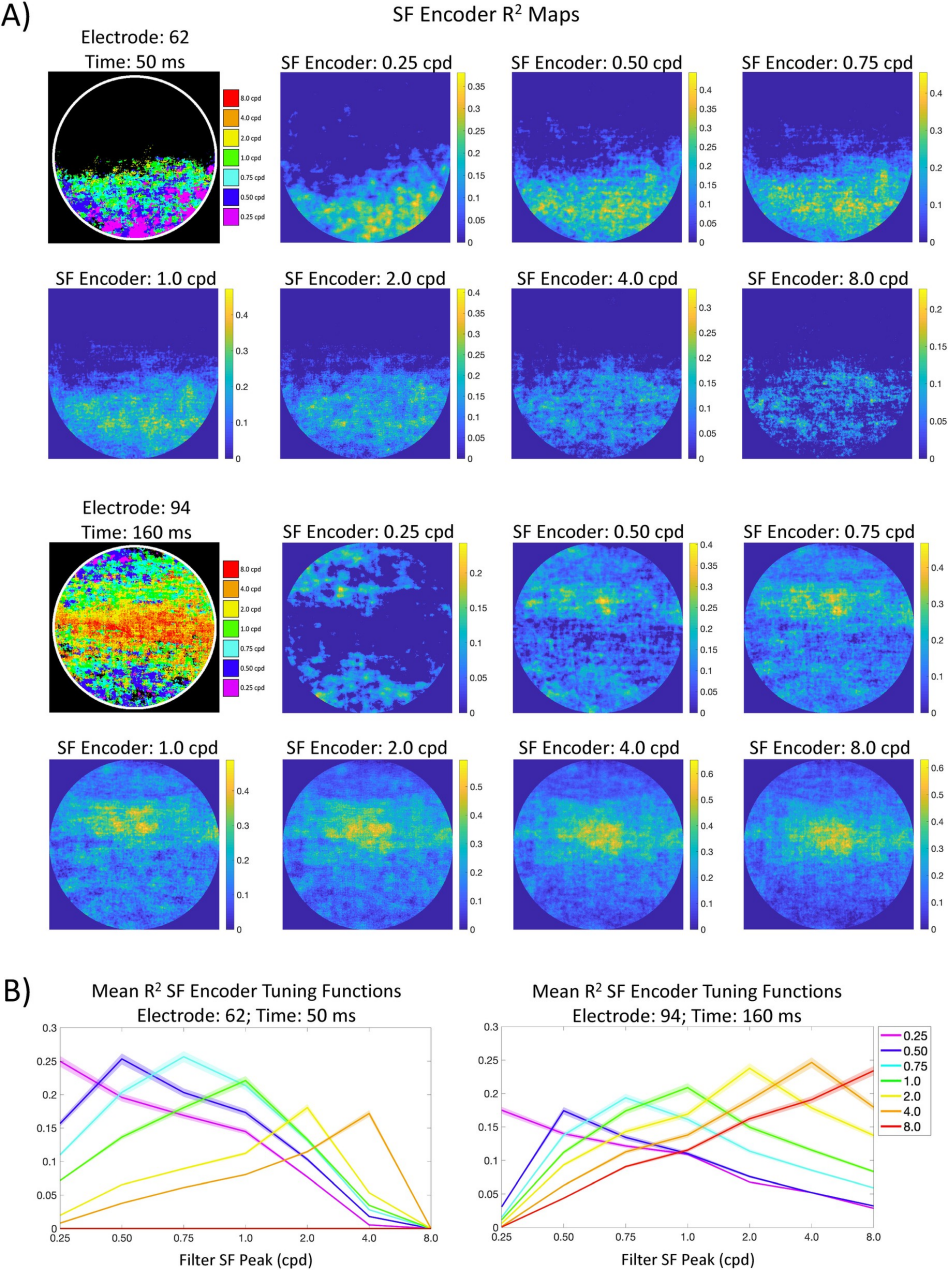
**A)** Example R^2^ maps from two different electrodes and time points. The DETI maps for each example are shown in the upper left of each set of R^2^ maps. Each R^2^ map shows significant R^2^s at each pixel location in image space. The color bar for each map shows R^2^. **B)** Example encoder R^2^ tuning functions for the two DETI maps shown in (A), averaged over all instances of each encoder’s tag in the DETI maps (y-axis is averaged R^2^, x-axis is encoder peak SF). The shaded region of each trace shows the 95% confidence interval over all instances of pixels for each encoder.

The linking operation serves to map each encoder’s peak SF to each pixel coordinate across a set of scenes to each encoder’s peak SF. First, each filter’s response at a given pixel coordinate across all images is first assembled into an 80 x 1 array (one filter response for each image as illustrated in **Fig 3**). Next, the natural log of each filter’s response array is regressed against the first PC at each 5 ms time step and electrode. This process is repeated on a pixel-by-pixel basis. The result of that procedure is a 2D encoder map for each of the seven SFs (for each electrode and time step), with each cell in a given map containing an R^2^ value (see **Fig 4A** for example encoder R^2^ maps). That value therefore provides an account of how encoder response variability across images (at any given pixel location) correlates with the variability of the first PC.

From here, two different mapping procedures can take place: 1) an image-general view of SF coding over the entire stimulus set, and 2) an image-specific view of the SF code for each scene in the stimulus set. Specifically, the first approach builds maps based on local (i.e., pixel-level) encoder variability across all stimuli at each time point (**Fig 3** lower right). Each pixel is ‘tagged’ with the encoder’s peak SF that best correlates with the first PC’s eigenvector. The second approach finds the encoder that best correlates with the first PC at each pixel coordinate within each stimulus, thereby providing scene-specific maps over time. Specifically, each pixel coordinate for a given scene is tagged with the encoder’s peak SF that had the shortest distance (smallest residual) between that scene and any one of the encoder regression lines across all images (**Fig 3** lower left). The code for the pipeline operations and both mapping procedures can be downloaded here https://pbsc.colgate.edu/~bchansen/HansenGreeneField2021/DETI_Code.zip.

### 2.0 Winner-take-all mapping validation

One of the more powerful features of the DETI mapping procedure is that it allows one to characterize the local transformational states of scenes in a space defined by populations of neural responses. By using a log-Gabor filter power encoder space, our primary goal was to assess which SF encoder best captured the local visual code at every pixel location across and within a large set of images and provide an account of how that local code changes over time (see Section 1.3). To achieve that goal, we reduced the encoder dimensionality at each pixel to that which accounted for the most variance in the VEPs (i.e., the peak SF of the encoder with the highest R^2^) and constructed the DETI maps based on that value (i.e., tagged each pixel with the encoder SF with the highest R^2^). While this approach may amplify the magnitude of the differential mapping, the goal of the DETI maps is to bring out those differences over time and electrodes. However, for that type of winner-take-all dimension reduction to be meaningful, there should be an underlying R^2^ tuning function in the encoder array at each pixel so that the highest R^2^ will capture the peak of the underlying R^2^ tuning function. Specifically, each pixel coordinate in a DETI map has only a single array of seven R^2^s associated with it. If there is no consistent underlying tuning structure (e.g., a distribution drawn from a random permutation of the images) across the seven R^2^s at all pixel coordinates, then a winner-take-all average of those arrays would be largely flat, with a single elevated point at the tagged SF, as opposed to a relatively gradual monotonic reduction in R^2^ magnitude with increased distance from the highest R^2^. We tested whether or not that was the case by averaging the R^2^ arrays across all pixels associated with each tagged SF for each DETI map separately at each electrode and time step. The result of that analysis showed clear evidence of R^2^ tuning (i.e., a gradual monotonic reduction in R^2^ magnitude with increased distance from the highest R^2^) for pixels tagged with any given encoder peak SF along with narrow confidence intervals. Example tuning functions are shown in **Fig 4B**, with the results from the comprehensive analysis shown in **S1B Fig**. Finally, to provide further support for this approach, we conducted the same tuning curve assessment analysis reported in **Fig 4** on another VEP dataset that was collected for another study that used different images than those used in the current study (see Materials & Methods) [39]. The results of that analysis (reported in **S2A Fig**) show tuning curve structure for each tagged set of pixels and are thus consistent with the results of the analyses reported in **Fig 4**.

### 3.1 Image-general DETI mapping results & mapping analysis

Prior to building the image-general DETI maps, all encoder R^2^ maps were corrected for multiple comparisons using the Benjamini-Hochberg procedure with a false discovery rate of 5% [40] across all pixels for each encoder separately at each time point and each electrode. We opted for that particular correction as it does a good job controlling for false positives while also being computationally efficient (computing DETI maps is very time intensive). As an added precaution, we ran permutation tests on the DETI mapping procedure. Specifically, the trial-averaged VEP data for each image was randomly shuffled for each participant (i.e., images were shuffled), and then averaged across participants. We then ran the DETI mapping procedure on that dataset and repeated that process 100 times (i.e., 100 simulated experiments). The resulting Benjamini-Hochberg corrected shuffled data maps over all electrodes at each time point were either completely empty (~94.5% over all electrodes and time points) or contained ~1.41% (median) pixels with encoder tags, demonstrating that this mapping procedure is largely resistant to noise. Using the same permutation tests, we found that the Benjamini-Hochberg correction remained robust to false positives out to a false discovery rate of 10% (~3.37% pixels with encoder tags).

Example DETI maps from participant-averaged data are shown in **Fig 1**. Please view the accompanying movie for the complete depiction of how different DETI maps evolve over time https://pbsc.colgate.edu/~bchansen/HansenGreeneField2021/HansenGreeneField_Figure1_Movie.mp4. **Fig 4A** shows example DETI maps for the image-general analysis. Here, the same participant-averaged data for two electrodes at two different time points are shown alongside the encoder R^2^ maps that were used to tag the encoder’s peak SF with the largest significant R^2^. **Fig 4B** shows the averaged R^2^ tuning curves as described in Section 2.0. The image-general DETI maps provide an opportunity to analyze and explore the general coding principles of scenes over time on an electrode-by-electrode basis. In the following two subsections, we first sought to further validate the mapping procedure by examining the extent to which the image-general DETI maps replicate the results of existing studies that used traditional VEP analyses to understand the neural dynamics related to real-world scenes (Section 3.1.1). In Section 3.1.2, we assess some of the general coding principles of scenes over time afforded by the image-general DETI maps.

#### 3.1.1 Relationship between the image-general DETI maps and traditional VEP analyses

**Fig 5:**
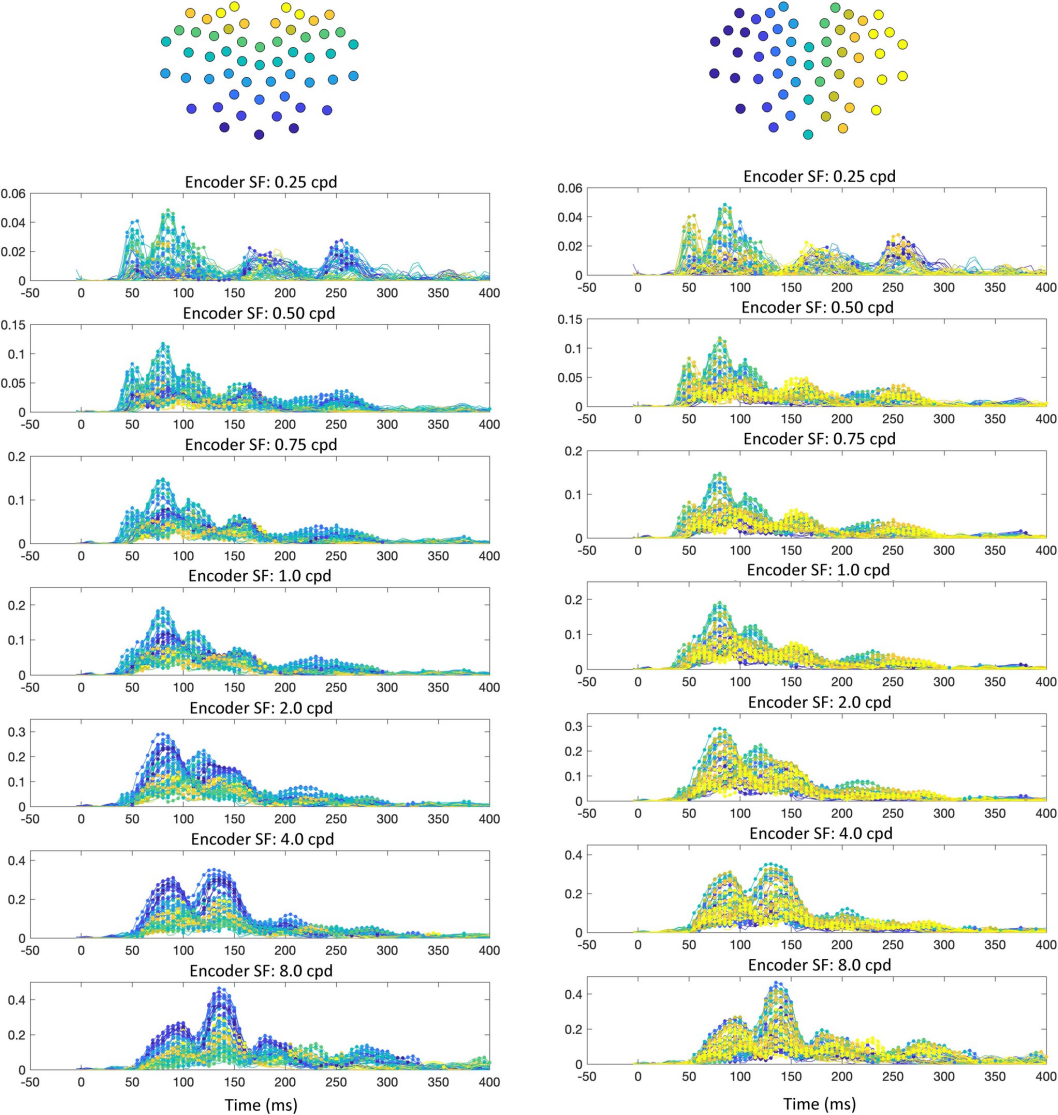
Encoder probability over time, averaged across participants. The y-axes show participant-averaged encoder probability (note that the axes are different across SF peak), with time (ms) on the x-axes. Each trace is from a specific electrode. Probabilities that were above the lower bound of 95% confidence intervals across participants are indicated with a marker point. The electrode traces are color-coded topographically in two ways (illustrated at the top of each set of plots). The left-hand plots are coded from ventral-posterior to dorsal-posterior portions of the scalp, with the right-hand side coded from left to right across the scalp.

The time-resolved maps shown in **Fig 1** (and the corresponding movie) reveal differences with respect to scalp topography and the relative contribution of SF encoders to each DETI map. Importantly, many of those differences are consistent with the results of traditional VEP analyses of real-world scene processing–namely those related to SF scalp topography and coarse-to-fine processing. To quantify those observations, we reduced the dimensionality of the DETI maps in several ways. To reduce the dimensionality from 206,643-pixel maps to a vector of seven SFs, we calculated the probability of observing pixels tagged with any given encoder’s peak SF by summing the number of pixels tagged by each encoder and divided each sum by the total number of visible pixels in the stimuli. This process was carried out for each electrode at each time step on a participant-by-participant basis. The resulting encoder probability-by-time calculations for each electrode and time step were then averaged across participants and shown in **Fig 5** (see **S3 Fig** for the results from the orientation analysis). Only probabilities where the lower bound of their 95% confidence interval (empirical confidence intervals computed over participants) did not cross zero are marked in that figure. The results plotted in **Fig 5** show that the posterior electrodes vary with respect to scalp location of the dominant encoder SF, likely reflecting their underlying calcarine sources in accordance with the cruciform model [41]. Specifically, the higher SFs (HSFs; 2–8 cpd) tend to dominate the DETI maps along the ventral-posterior electrodes, with the lower SFs (LSFs; 0.25–0.75) dominating the DETI maps along the dorsal-posterior electrodes–both observations are consistent with previous literature [42]. We verified that observation by averaging the probabilities for the ventral electrodes and dorsal electrodes (separately) for each participant and conducted a time step by time step paired samples t-test. The results of that analysis showed statistically significant differences between ~48 ms and 149 ms (all P’s < 0.05, corrected for multiple comparisons by setting the p value to the proportion of t values produced by 1000 permutations that were above the observed t values). The extent of SF lateralization shown in **Fig 5** is less clear, but the lowest SF does tend to be somewhat right lateralized between 52 ms and 108 ms (paired samples t-test, all P’s < 0.05, corrected for multiple comparisons as stated above), which is consistent with the fMRI literature [43], though caution must be used in further consideration of that result due to the poor spatial resolution of EEG.

**Fig 6:**
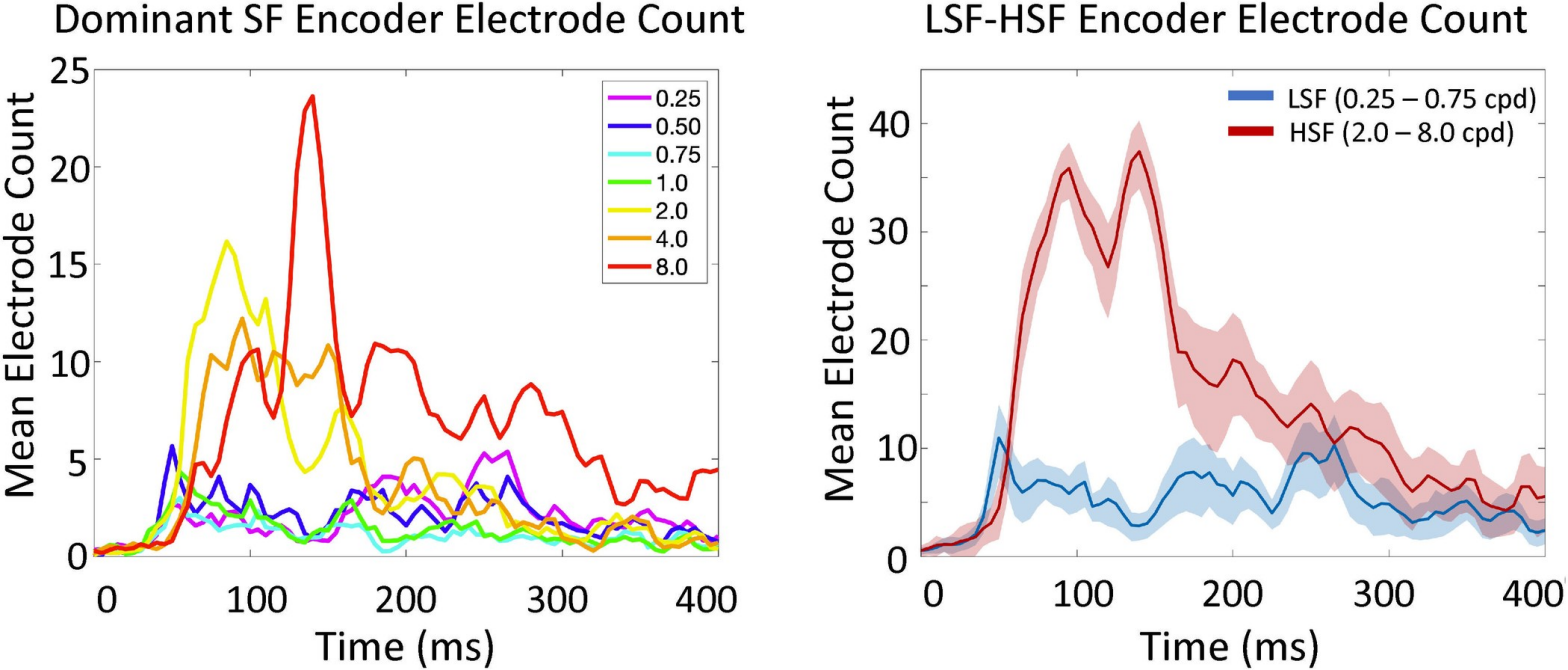
Participant averaged electrode counts based on encoder dominated DETI maps. The left-hand plot shows the number of electrodes that were dominated (largest sum) by each of the seven encoders. The right-hand side is a summary of that plot and shows the electrode counts summed over LSFs (0.25–0.75 cpd) and HSFs (2.0–8.0 cpd). The shaded area shows the 95% confidence intervals over participants.

The time-resolved maps shown in **Fig 1** (and the corresponding movie) also show evidence for coarse-to-fine processing [44–45] whereby the LSFs tend to tag DETI maps across electrodes more frequently before the HSFs (e.g., compare the posterior electrode montage at 50 ms and 160ms in **Fig 1**). To test that observation with the data plotted in **Fig 5**, we averaged those data across electrodes for the LSFs and HSFs (separately) for each participant and conducted paired samples t-tests time point by time point. The results revealed that the LSFs dominate between 40 ms and 55 ms (all P’s < 0.05). Another way to visualize the SF probability differences over time is by electrode density associated with each encoder’s SF. To do that, we tagged each electrode with the encoder SF that was most prevalent in its map and summed the number of electrodes dominated by each encoder at each time point (**Fig 6**). The results show that LSFs dominate early (~50 ms), followed by the HSFs, with the 2 cpd and 4 cpd encoders preceding the 8 cpd encoder. Interestingly, every time point yields electrodes that are dominated by different encoder SFs, revealing a multiscale representation of scenes over time. In other words, every SF that we explored is explaining VEP variance simultaneously across the posterior scalp, though the HSF neural sources tend to dominate the scalp.

Finally, the results from the orientation-based encoders do not show a clear topographic organization, but instead show that orientations at and near horizontal have the lowest probability in the maps over time (**S3 Fig**). Such a result is consistent with normalization operations in visual cortex and scene selective cortices that operate to reduce the magnitude of horizontal information [46–47].

The consistency between the image-general DETI mapping results and existing results from traditional VEP analyses provides an important validation for the DETI mapping approach and shows that this procedure can successfully map the SF scalp topography without resorting to multiple experiments that present stimuli at different locations in the visual field to avoid dipole cancellation. Further, this approach provides a *much* more detailed account of the code that underlies the topography with full images that span a sizable portion of the visual field.

#### 3.1.2 The general coding principles of scenes over image space across time

**Fig 7:**
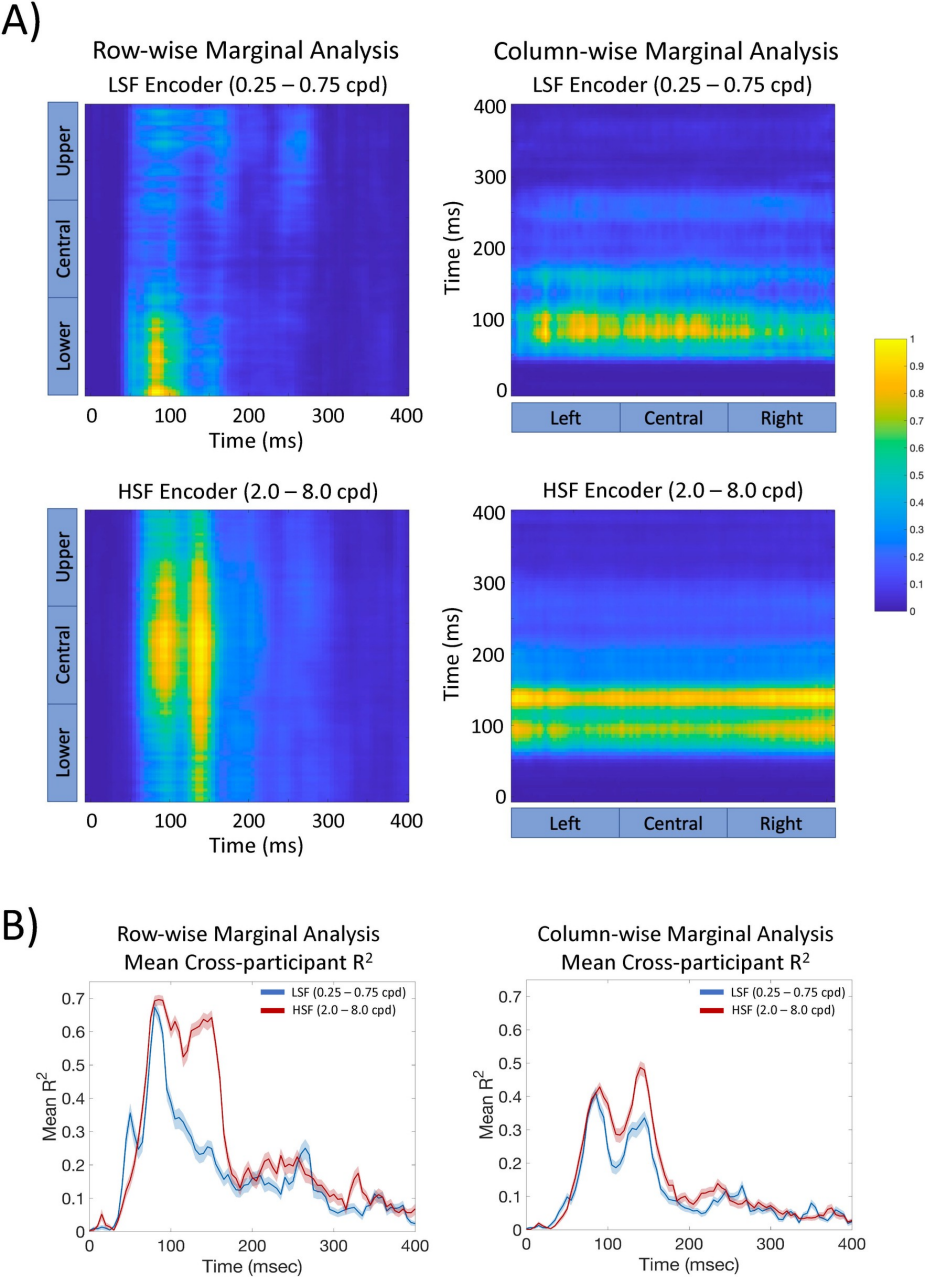
**A)** Marginal analyses of the 2D DETI map histograms for the lower (LSF) and higher (HSF) encoder peak SF. The row-wise marginal analysis consists of an average across the histogram maps from left to right for each time point. The columns of that plot were first normalized over time and then normalized again within each column to emphasize encoder fit magnitude over time and space. The column-wise marginal analysis was carried out the same way, but from top to bottom of the R^2^ maps (the normalization therefore took place row-wise). For a dynamic view of each marginal analysis, follow this link https://pbsc.colgate.edu/~bchansen/HansenGreeneField2021/HansenGreeneField_Figure4_Movie.mp4. **B)** Averaged cross-participant histogram maps for each column in the row-wise marginal analysis (left) and each row of the column-wise marginal analysis (right). The shaded region shows the 95% confidence interval across participants.

Having validated DETI mapping through the replication of existing results of scene processing obtained with traditional VEP analyses, we turn our attention to what this approach can tell us about how general scene regions are coded over time. Towards that end, we analyzed DETI map variation over all electrodes at each time point. Because cortical folding varies from person-to-person (which influences how VEP signals register on the scalp), we could not simply collapse across participants at corresponding electrodes. Instead, we split each electrode’s fully tagged DETI map into a set of seven binary maps, one for each encoder, and then summed those maps (pixel-by-pixel) across all electrodes at each time point. That process results in 2D histograms for each encoder, which were then summed across participants. To provide a comprehensive view of the spatial biases in encoder maps over time, we again grouped the lower SF encoders (0.25–0.75 cpd) and higher SF encoders (2.0–8.0 cpd) and conducted an upper-to-lower and left-to-right marginal analysis on the LSF and HSF maps at each time point (**Fig 7A**). To assess participant agreement between the different marginal analyses, we regressed the upper/lower and left/right biases at each time point across participants (**Fig 7B**).

The results shown in **Fig 7A** reveal that the upper and lower portions of images are coded by LSFs, with the HSFs coding the central portion of images. These biases were largely consistent across participants, especially during the earlier time window (e.g., 50–180 ms) (**Fig 7B**). Interestingly, those spatial biases are not static, but vary in a nonuniform manner over time. Specifically, in the row-wise marginal analysis, the lower portion of images is dominantly coded early (~50 ms) by LSFs, with the upper portion of image space dominating later (~250 ms), again with LSFs (which is also apparent in the movie associated with **Fig 1**). The central portion of image space shows two waves of HSFs, with the first wave more centralized than the second. When compared to **Fig 6**, the first wave corresponds to 2–4 cpd, with the spatially broader wave corresponding to 8 cpd. The column-wise marginal analysis revealed additional asymmetries that were observed laterally across images with LSFs showing an early (~70 ms) central-to-left hand bias, with HSFs showing a slightly later (~80 ms) bias toward the right and far left portions of images. The left-right asymmetries are consistent with the fMRI literature [43] that reports a right-lateralized occipitotemporal LSF bias (which would show up here as a bias on the left), and a left-lateralized temporal HSF bias (which would show up here as a bias on the right). Lastly, we conducted a similar analysis using the raw R^2^ maps from each participant. The results of that analysis are largely consistent with the spatiotemporal patterns revealed by the marginal analyses conducted on the 2D histograms (see **S4 Fig**), as well as the encoder probability over time analysis (see **S5 Fig**) and are largely replicated using the additional dataset mentioned in Section 2.0 and reported in **S2B Fig**).

Crucially, the nonuniformities over image region and time would not be expected from a simple linear model based on retinotopic mapping of SF preference and suggest that the neural code for different image regions changes SF preferences differently over time, thereby providing insight into a possible prioritization of different image regions as time advances. For instance, an early prioritization of the ground plane may support rapid judgements regarding scene navigation [48–49], with a later upper image region analyses focused on landmark organization [50].

### 3.2 Image-specific DETI mapping results & mapping analysis

In addition to providing useful insights into how image regions are coded across a set of images, the DETI mapping procedure offers the ability to examine the local code for individual images over time. All image-specific mapping reported here was based on the regression fitting procedure that was carried out for the image-general analysis, but here was focused on mapping based on minimal residual error from each encoder’s regression line (refer to **Fig 3**). The random permutation analysis reported in Section 3.1 suggests that the Benjamini-Hochberg correction procedure tended to be overly conservative, so we adjusted the false discovery rate to 10% for the analyses reported here (the results from analyses with a 5% false discovery rate were largely consistent with those reported here and are shown in **S6 Fig**).

**Fig 8:**
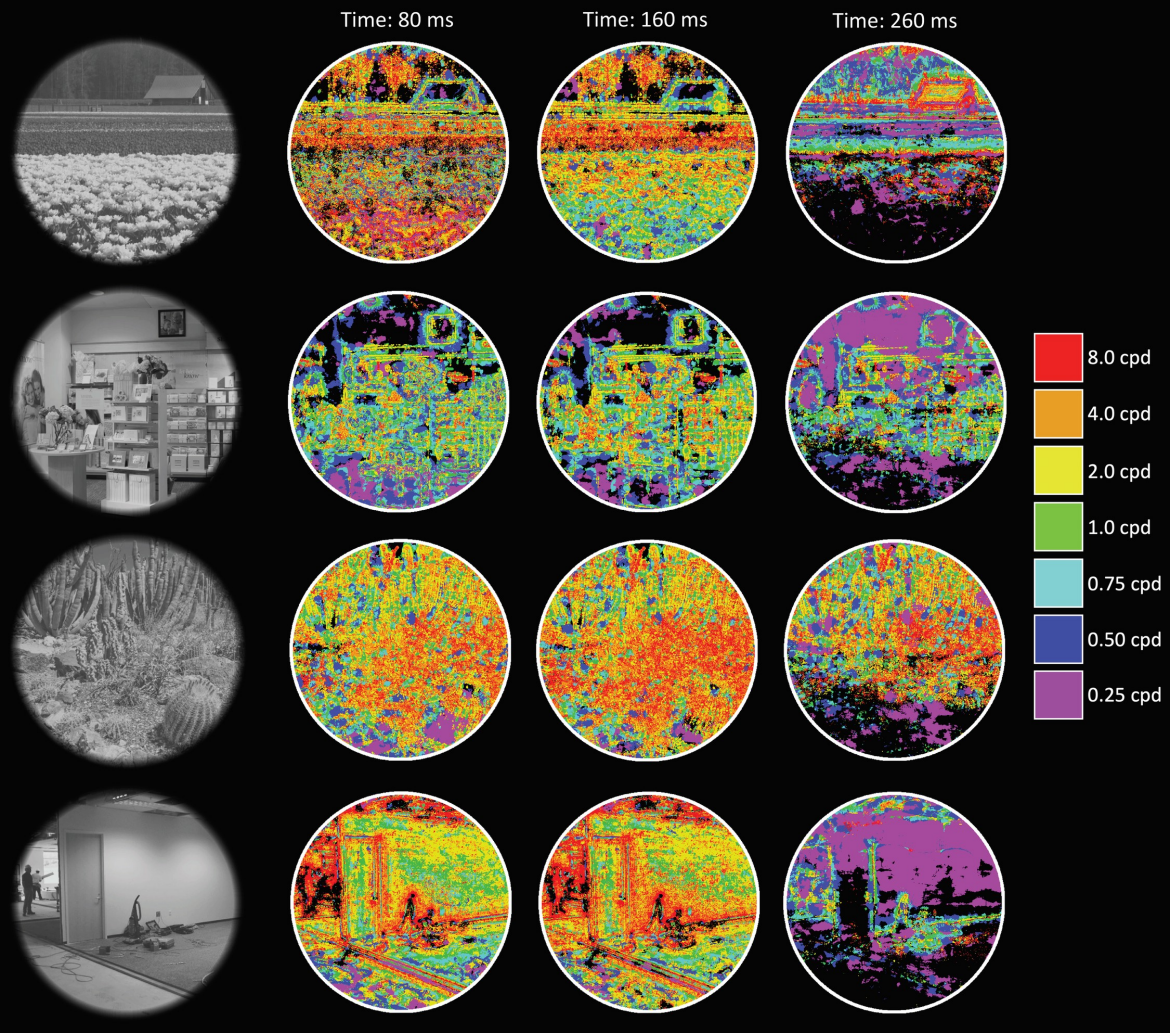
Additional stimulus examples and their DETI maps at different time points from electrode 90.

Example image-specific DETI maps from participant averaged data are shown in **Fig 2**. Please view the accompanying movie for the complete depiction of how different DETI maps evolve over time https://pbsc.colgate.edu/~bchansen/HansenGreeneField2021/HansenGreeneField_Figure2_Movie.mp4. Additional image-specific map examples are shown in **Fig 8**. Together, **Figs 2** and **5** (and the corresponding movie) illustrate some of the diversity of image transformations over time.

**Fig 9:**
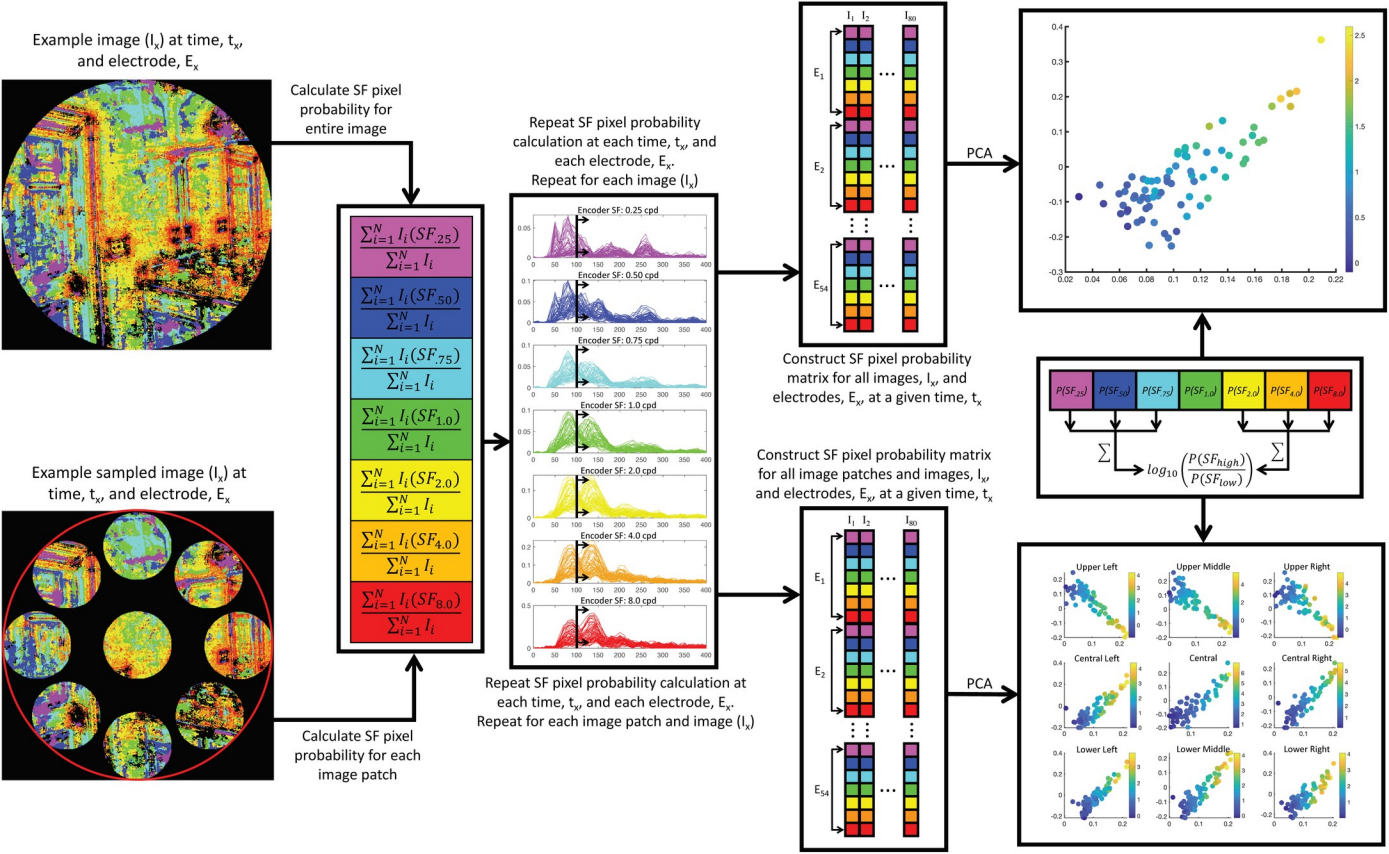
Illustration of the procedure for projecting all encoder-tagged images into a state-space representation at each time point for each participant using all electrodes. This procedure was either carried out for entire images (top left) or within patches located at nine different regions of the images (bottom left). Encoder probabilities were calculated as described in the text for whole images or image patches. At each time point and across all electrodes, each encoder’s probability was stored in a matrix as an array on an image-by-image basis (e.g., a 378 x 80 matrix for each time point). Each matrix was then submitted to PCA, with the first two PCs defining the encoded image’s state-space. Each image was then characterized by taking the log_10_ HSF-to-LSF probability ratio (see text for further detail).

While the image-general maps revealed the general coding principles across images for our set of stimuli, here we analyzed the image-specific maps to provide an account of the various encoder-based transformations that each scene undergoes over time. To do that, we first constructed a summary statistic (illustrated in **Fig 9** and described later in this section) that would characterize the transformational state of each image at each time step. Doing so would enable us to track (over time) where any given image is in the neural response space. To that end, we first built a low-dimensional DETI map space to plot each image and then tested how well our summary statistic predicted the location of each image in that space. The results of that analysis allowed us to proceed with that statistic in subsequent analyses of the transformational states of images over time.

**Fig 10:**
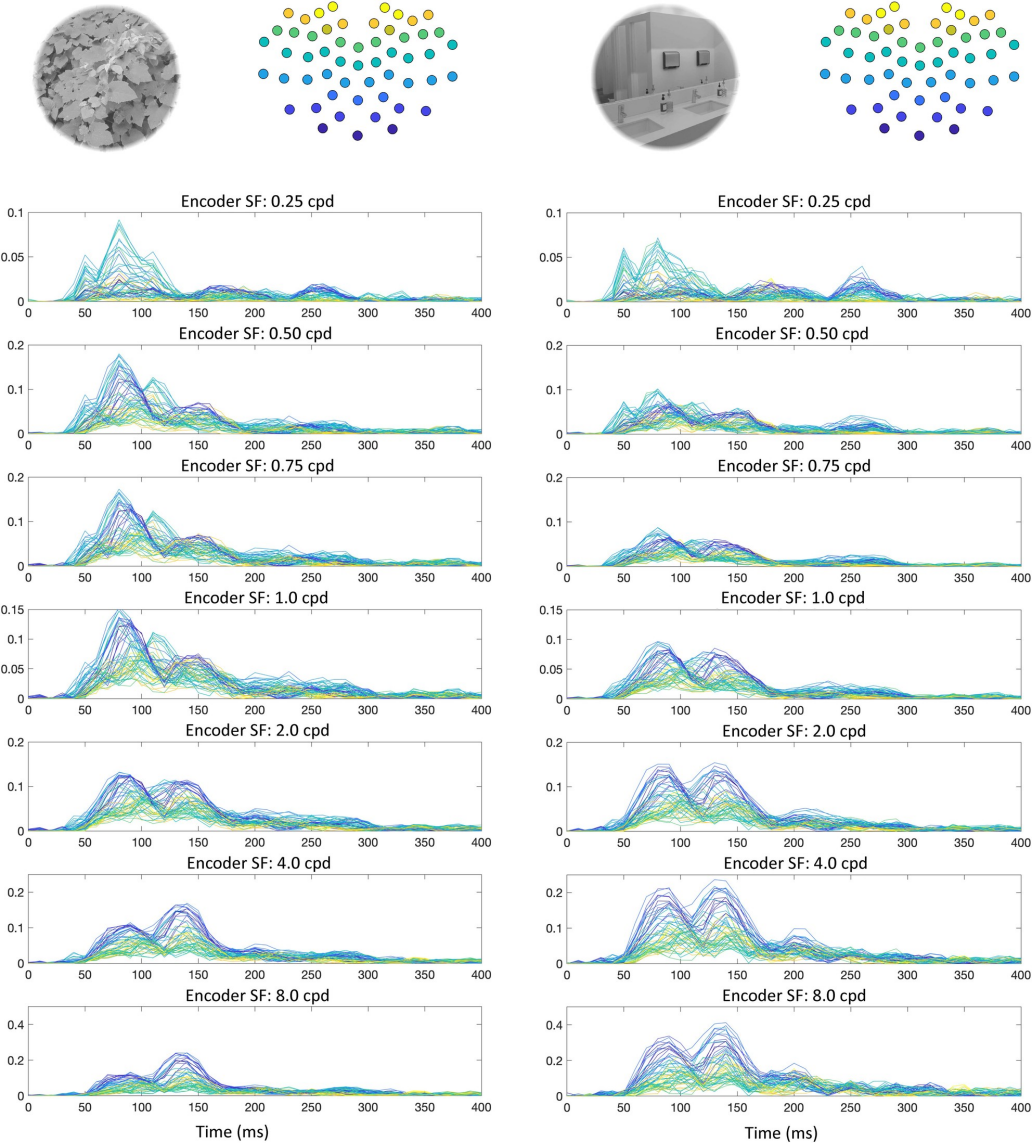
Encoder probability over time for two stimulus examples (upper left of each set of plots), averaged across participants. The y-axes show participant-averaged encoder probability, with time (ms) on the x-axes. Each trace is from a specific electrode and color-coded from ventral-posterior to dorsal posterior electrode (illustrated at the upper right of each set of plots).

To build a low dimensional image-specific DETI map space, we chose to reduce the dimensionality of those maps by calculating the probability of observing pixels tagged with any given encoder’s peak SF, thereby reducing any given image-level map to seven probability values (similar to the image-general analysis). That process was carried out for each electrode and time step on a participant-by-participant basis. Example probabilities (averaged across participants) for each encoder over time and electrode for two example images are shown in **Fig 10**. Consistent with the image-general mapping, individual images exhibit a tendency for HSFs to dominate the electrode maps along the ventral-posterior electrodes, with LSFs dominating the electrode maps along the dorsal-posterior electrodes. Importantly, each image showed a relatively unique probability-by-time ‘signature’ between and across electrodes. For example, the image on the left-hand side of **Fig 10** shows an early dominant multimodal distribution for lower SF which gives way to a weaker bimodal HSF distribution. The image on the right-hand side shows a much weaker multimodal distribution at the lowest SFs which changes to a dominant bimodal distribution for SFs > = 0.75 cpd.

**Fig 11:**
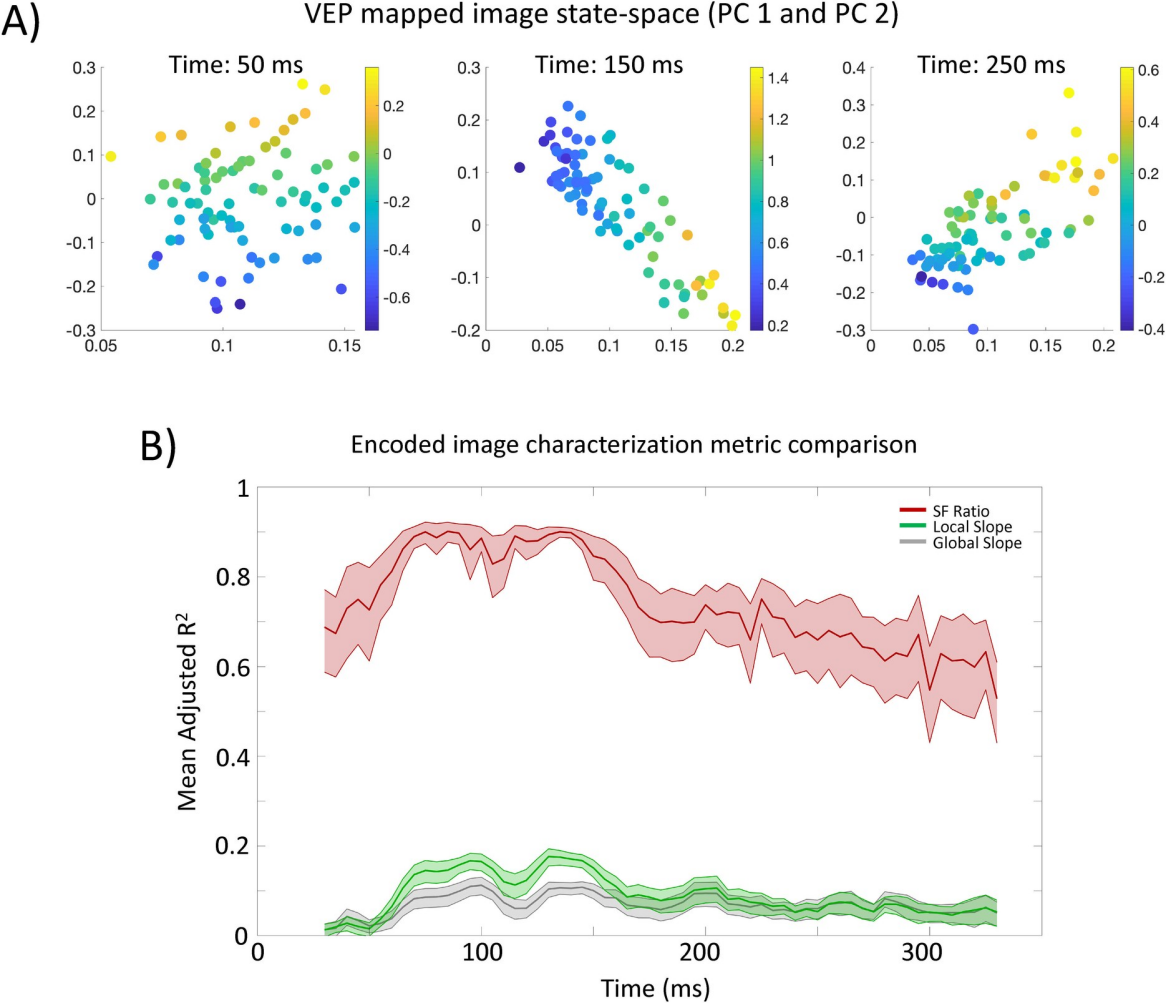
**A)** Principal component (PC) defined VEP-mapped image state-space from an example participant at three different time points (refer to text and **Fig 9** for further details). Data points are images, and the color bar corresponds to the log_10_ HSF-to-LSF probability ratio for each image in that space. The x- and y-axes are PC 1 and 2 respectively. **B)** Characterization metric comparison for explaining the relative location of each image in PC-defined state-space over time. The y-axis shows the average adjusted R^2^ across participants. The shaded region shows the 95% confidence interval of the fits across participants.

With the probability-by-time data in hand, we next projected all image-specific DETI maps into a state-space representation based on all electrodes at each time point for each participant. Briefly, for any given time point, each encoder’s probability for each electrode was stored in an array on a map-by-map basis, resulting in a 378 x 80 matrix for each time point (i.e., 54 electrodes * 7 encoders X 80 scenes) (see **Fig 9**). Thus, each image’s map is represented as patterns of SF probability across encoders and electrodes. Including all 54 electrodes allowed representations based on visual signals from different regions of the of the calcarine sulcus [41–42]. The resulting matrix was then submitted to time-resolved PCA (with electrodes as observations and scenes as variables). The first two PCs accounted for 87.4% (median) of the variance across time, images, and participants, with the first PC accounting for 80.7% (median). All images could then be represented in a 2D encoder probability state-space defined by the first two PCs at each time point and participant. Examples of that space for a given participant at three different time points are shown in **Fig 11A** and show how images are organized in the low-dimensional DETI map space.

Next, we sought a summary statistic that could characterize the relative positioning of the images in the DETI map space according to encoder probability, thereby enabling us to ‘track’ the different transformational states of any given image. Specifically, we characterized each image by the log_10_ ratio of the summed HSF probabilities (2.0–8.0 cpd) to the summed LSF (0.25–0.75 cpd) probabilities (an SF probability ratio) at each time point as depicted in **Fig 9**. Characterizing images according that summary statistic defines any given transformational state as the relative amount of the image coded by HSFs vs LSFs. To assess the ability of that characterization to explain the first two PCs, we submitted the SF ratio for each image (and at each time point) to multiple regression and show the adjusted R^2^ over time in **Fig 11B**. The result of that analysis shows that SF probability ratio provides an excellent account of the relative positioning of images in the DETI map state-space for all participants. We also compared the SF probability ratio to that which could be explained by simple Fourier image statistics based on: 1) the amplitude spectrum slope of each image, and 2) the ratio of local filter response slopes (shallow slopes to steep slopes) at each pixel (also shown in **Fig 11B**). While the Fourier image summary statistics were successful at explaining image position in that space, the SF probability ratio is far superior, meaning that the different transformational states that the images undergo are only minimally explained by simple Fourier image statistics.

**Fig 12:**
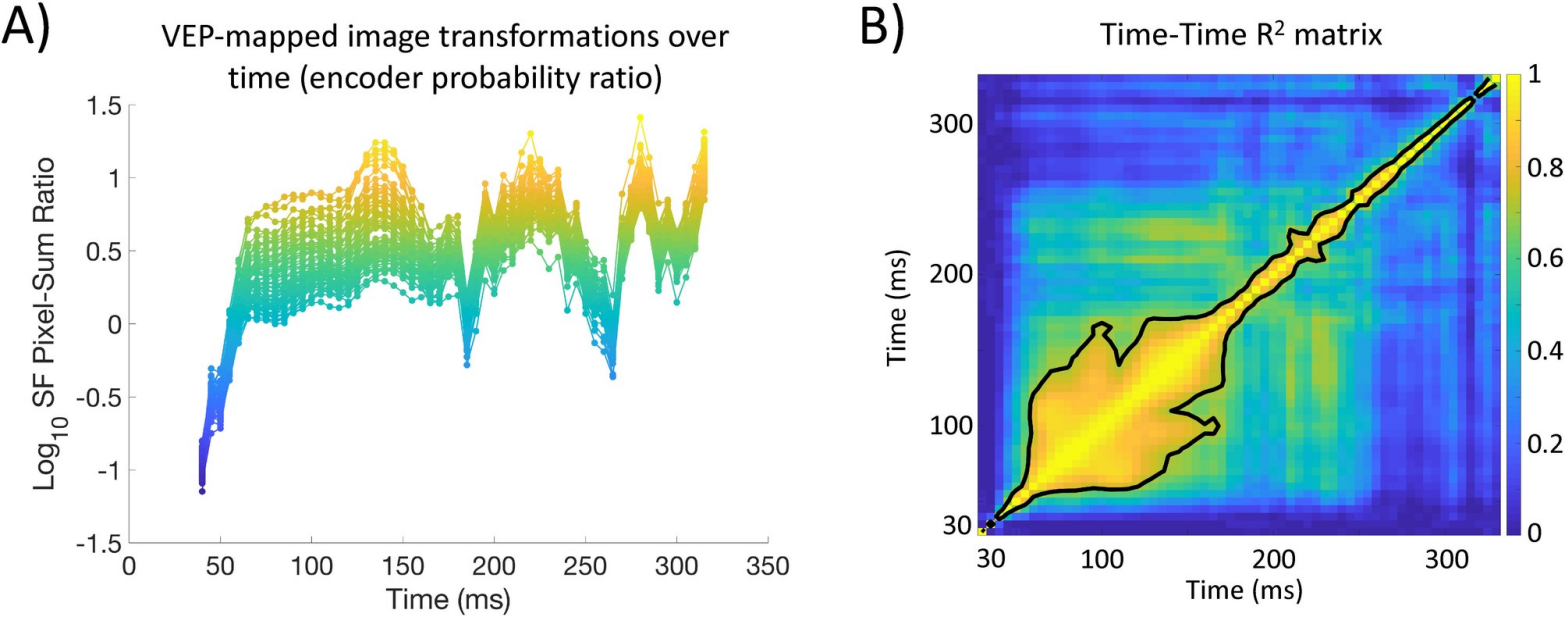
**A)** Participant averaged log_10_ HSF-to-LSF probability ratio for each encoder tagged image over time across the entire stimulus space (i.e., all pixels). **B)** Participant averaged time-time R^2^ matrix assembled by regressing the log_10_ HSF-to-LSF probability ratios across images for each time point against every other time point (color bar is R^2^). The contour line encapsulates the top 12% of the R^2^s.

The success of our summary statistic enabled us to visualize each image’s transformation over time. **Fig 12A** plots the participant-averaged SF probability ratio at each time step for each of the 80 images and illustrates how the relative HSF to LSF coding of images changes over time. The most prominent variation in **Fig 12A** is an interesting, possibly two-stage [51–53] pattern of transformations in that the images first show an initial LSF based code (~50 ms), followed by a relative HSF code (~70 ms to ~140 ms) where the encoder SF ratio variance is ~3 times larger than any other time window. Around 150 ms, the image transformations undergo what appears to be intermittent LSF transformations at ~180 ms and ~260 ms, possibly indicative of recurrent processes [52–53]. The variation of the SF probability ratios over time was verified with a one-way ANOVA including each image’s ratio and factor of time, F_(55,1288)_ = 2.7, P < .001. Because the SF probability ratio characterization provides a strong account of where each image is located in the DETI map state-space, we next sought to assess the stability of the *relative* positioning of the images in that space over time. That is, we aimed to assess the relative similarity of the transformational states of images over time thereby testing the relative persistence of those states over time. To do that, we ran a time-time regression analysis on each participant’s SF probability ratios. Specifically, we regressed the ratios of all images at any given time point against the ratios at every other time point, and then averaged the resulting R^2^s across participants (**Fig 12B**). The results of that analysis show an early period (~70 ms to ~160 ms) of relative stability, meaning that the transformational states of the images during that window of time covary with one another. However, after that window of time, there is an increase in the temporal variability (which can be interpreted as a reduction in temporal similarity) between transformational states, suggesting that each transformation becomes more unique as time advances past ~160 ms.

**Fig 13:**
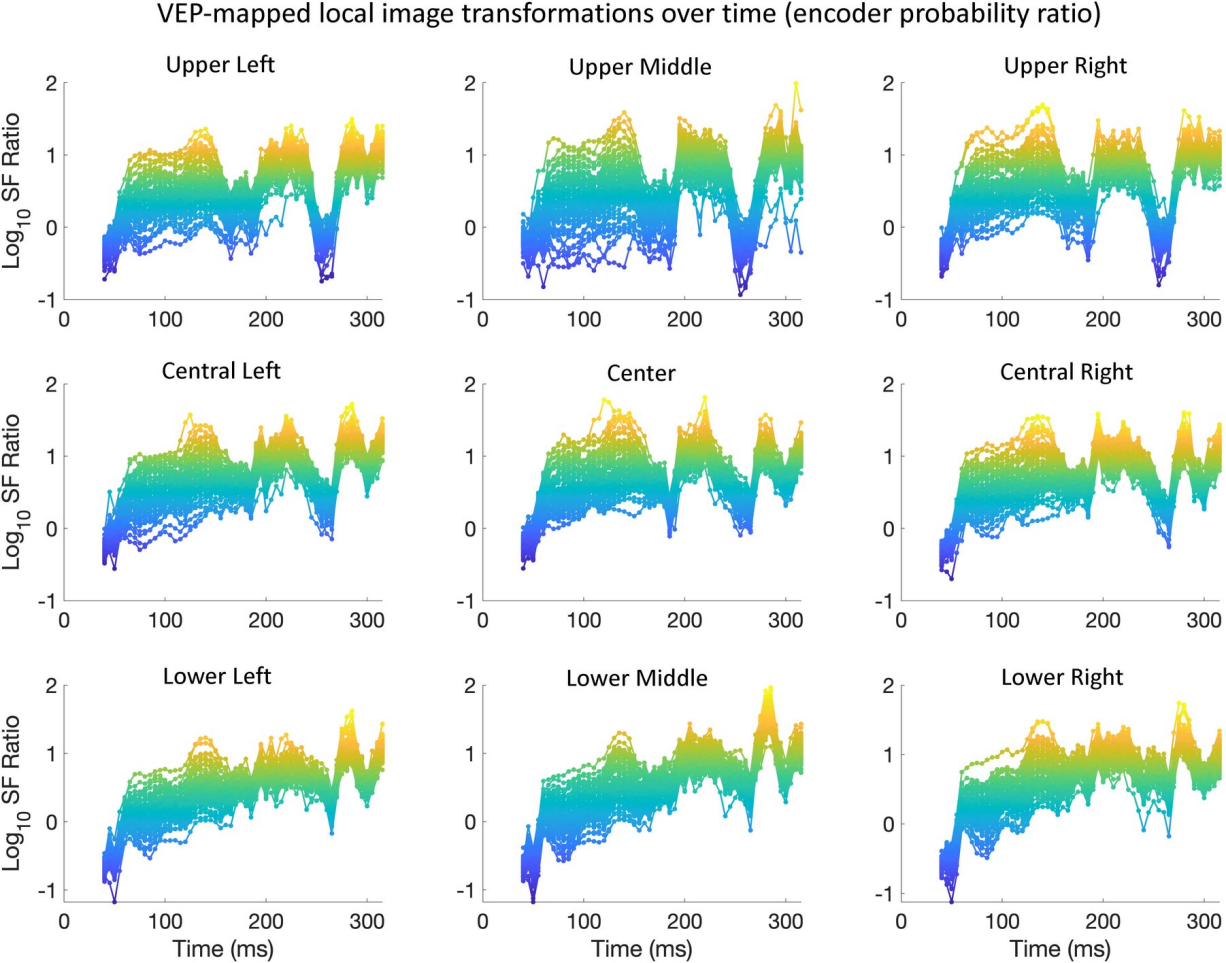
Participant averaged log_10_ HSF-to-LSF probability ratio for each encoder tagged image over time, measured at nine different locations within stimulus space (see text for more details). The format of the plots is the same as in **Fig 12A**.

While the results reported in **Fig 12** provide insight into the different transformational states (as defined by SF probability ratios) that scenes undergo, they do not provide an account of how local image regions shape those states. Understanding local variation is important because as receptive field size increases along the visual processing stream, image coded by smaller receptive fields likely contribute differentially to larger receptive fields that shape high-level representations. Understanding the local variation therefore offers some insight into how different image regions contribute to the higher representations. We therefore focused on an assessment of the transformations within localized image windows. Out of practicality, we ran this analysis within nine different spatial windows using a polar coordinate system to define the position of each spatial window (refer to **Fig 9** for an example). Each spatial window had a diameter of 128 pixels (4.7° of visual angle). The center-to-center distance between the center window and the outer windows was 190 pixels (7.1° of visual angle), with the center-to-center distance between each outer window being 160 pixels (6° of visual angle). The analysis described above to produce **Fig 12A** was carried out on each window location across all images on a participant-by-participant basis and then averaged across participants (**Fig 13**).

**Fig 14:**
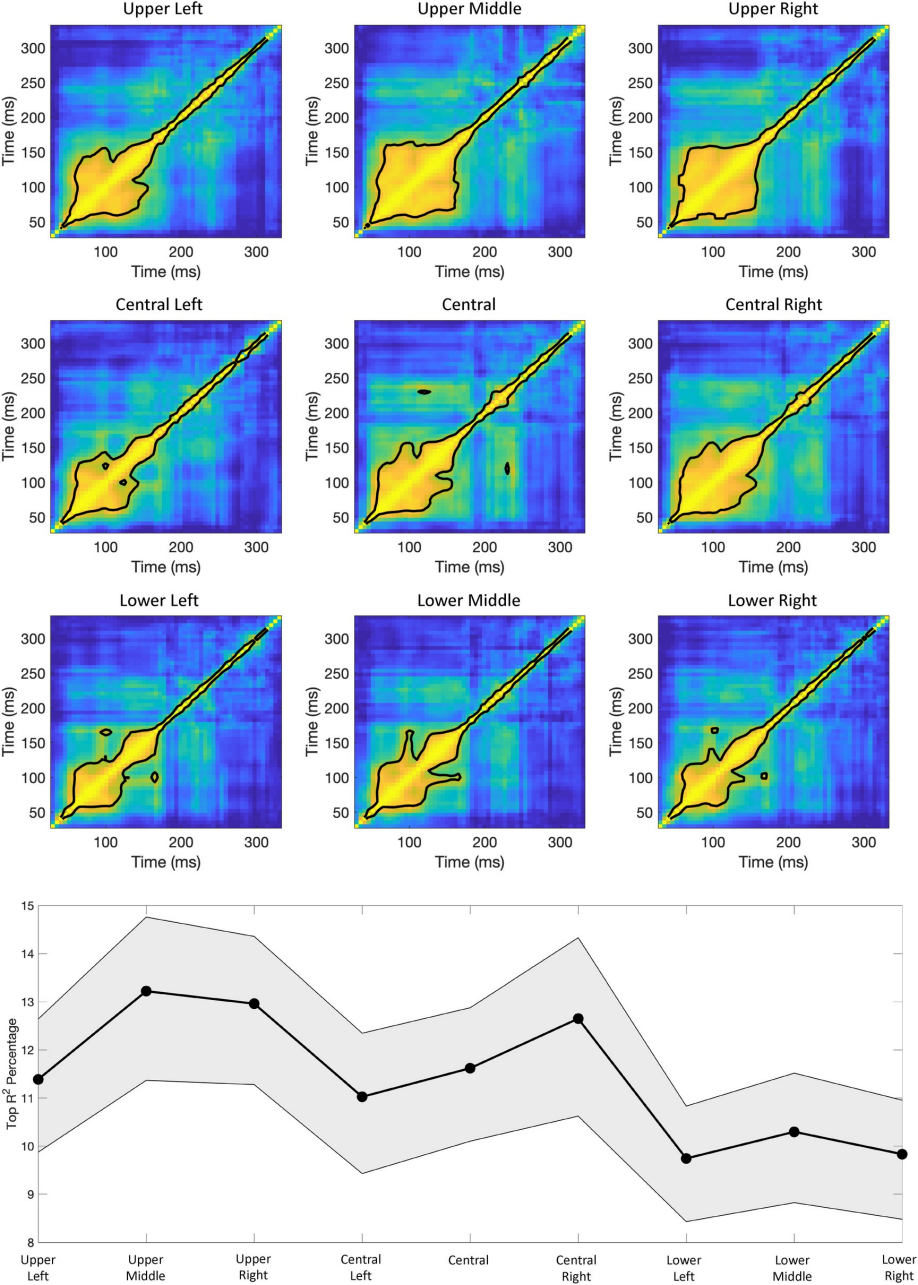
Top: Results from the time-time regression analysis on the participant averaged SF probability ratios within each of the nine local image windows. Bottom: The percentage of time-time R^2^s that fell within the top 12% contour area was calculated for each participant, and then averaged across participants (gray area shows the 95% confidence interval).

**Fig 13** reveals that different image locations undergo a relatively unique transformational process over time, with the largest difference being between the upper and lower portions of the image, with the central portion being somewhat intermediate. Specifically, the upper and central portions of different images show transformational states over time that are similar to that found with the whole image analysis (**Fig 12A**), with the lower image regions showing transformational states that follow a monotonic rise from LSF-based representations to HSF-based representations. To assess the relative similarity of the transformational states of the nine image regions over time, we ran the same time-time regression analysis mentioned before on the SF probability ratios for each region (**Fig 14**). The results of that analysis were somewhat similar to the whole image analysis (**Fig 12B**), with the upper and central portions of image space showing the gradual reduction of similarity between transformational states over time. On the other hand, the lower portion shows overall less similarity between transformation states. That plot illustrates the tendency of the lower image windows to result in more temporal variation, thereby suggesting more unique transformational states on a time point by time point basis. That observation was first verified with a one-way ANOVA including each image’s ratio and factor of time, F_(8,207)_ = 2.9, P = .005, with post-hoc t-tests showing significant differences between all lower windows and the upper middle, upper right, and central right windows (P’s < .02). This suggests more dynamic representations in the lower portion of image space compared to the upper portion and may reflect a shift from a higher-dimensional representations to lower-dimensional representations across the image that may be relevant for perceptual decision making [54]. Further, such a differential shift in dimensionality across the image may suggest a need to maintain different degrees of spatial dimensionality to allow for flexibility in perceptual decision making (though such an account is in need of further research). Nevertheless, what is striking about this local analysis is that the representations contained within each region (upper, central, lower) is more similar than the representations between each region. This further supports the finding that there are different neural coding operations at different image locations over time. Importantly, the differential transformational states within different image regions suggest that the temporal coding of visual information is far more complex than a simple coarse-to-fine analysis and subsequent mapping to higher cortical representations [44–45].

**Fig 15:**
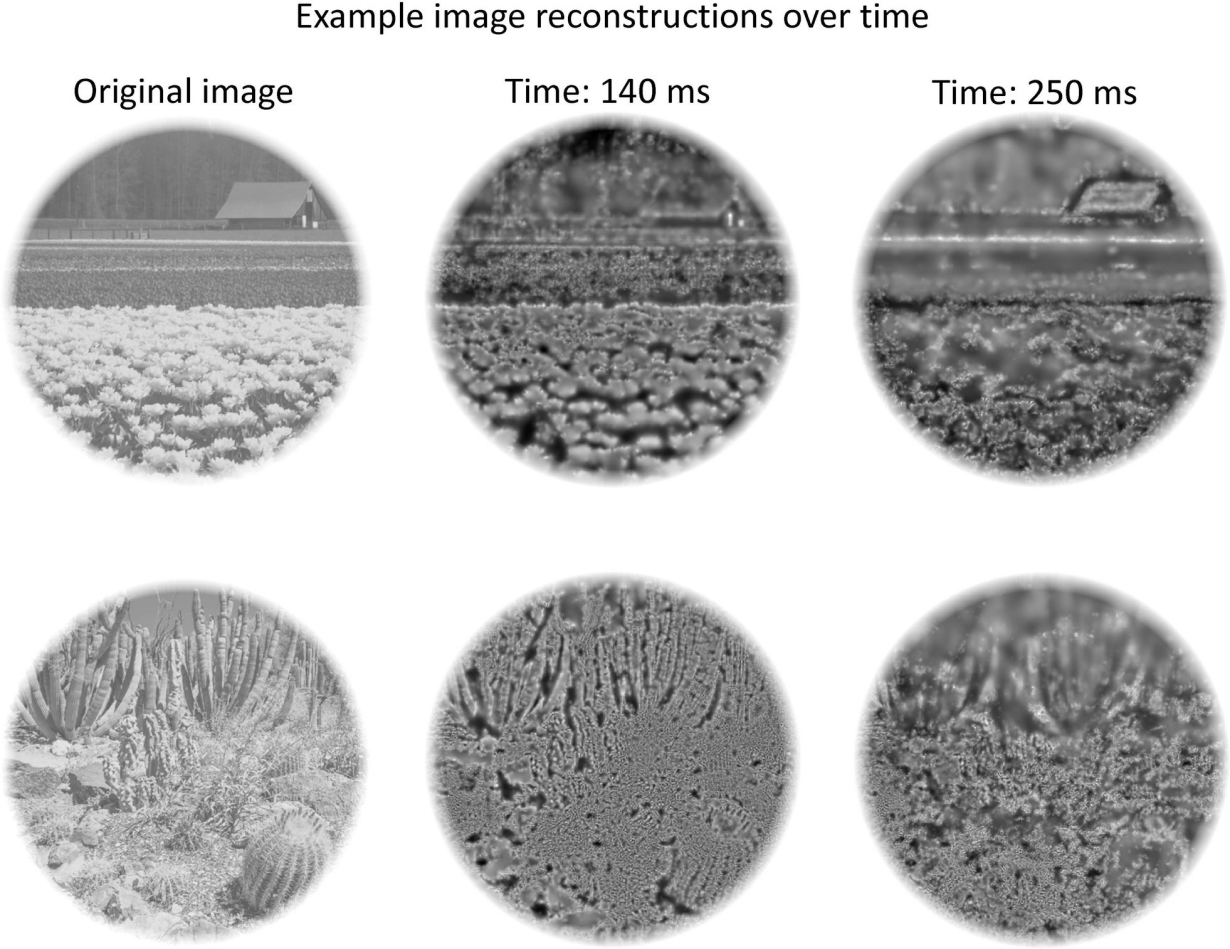
Example stimuli along with their SF filter reconstructions based on the 2D encoder histograms at two time points (see Materials & Methods for further details).

Finally, to provide an approximate visualization of how the images may appear in different transformational states, we ‘reconstructed’ example images using an approach that is similar to the SF bubbles technique in the spatial domain [55] (see Materials & Methods for details). The examples shown in **Fig 15** show two time points, with an earlier time point (140 ms) showing the relatively constant SF mapping across image region, with a later (250 ms) inconsistent representation across image region where the upper portion of the images are in an LSF transformational state and lower portion of the images are in an HSF transformational state.

## Discussion

The DETI mapping procedure offers many advantages over traditional EEG component analyses by providing a framework to assess how each image region contributes to the underlying VEP signals. Specifically, electrodes over the posterior scalp tend to contain signals that carry information in the upper or lower peripheral visual fields, while electrodes over the occipital pole tend to signal for information in the central visual field [41–42]. However, using raw VEPs to assess the entirety of the visual signal simultaneously for large-field scenes yields overlapping components that differ in polarity as a function of visual field location. What that means is that the complete spatial representation of the visual signal would be largely obscured by dipole cancellation [41]. The DETI mapping procedure circumvents this problem by relying on low dimensional signal *variance* to map VEPs to scene images in encoder space. Consistent with existing encoding (and decoding) analyses applied to time-varying neural signals [14,21,39,56–59], the DETI mapping procedure emphasizes a departure from an activation-based analysis to more of an information-based analysis based on the variance of time-varying neural signals. However, the mapping of VEP variance to our encoder space differs from existing multivariate approaches in that it is strictly a mass-univariate encoding approach that maps VEP signal variance to the image domain on a pixel-by-pixel basis, thereby enabling a relatively clear visualization of the early transformational states that scenes undergo. Further, the electrode-by-electrode mapping provides opportunities to evaluate the early transformational states of neural signals that originate at different locations along the calcarine sulcus [36,41–42,60] (though the maps can be submitted to a multivariate analysis, as was done here and illustrated in **Fig 9**). In fact, DETI mapping provides a rich dataset (54 electrodes X 206,643 pixels X 7 encoders = 78,111,054-dimensional space per time point) for a variety of analyses, thereby enabling multiple levels of downstream mapping of the early visual code. For example, the relative transformational states between scenes and scene regions can be used to project DETI maps into other lower-dimensional spaces for representational similarity analysis (RSA) at each time point. Such an analysis would allow insight into how different transformational states of scene regions map onto the knowledge structures that drive intelligent behavior [11–15]. Lastly, because DETI mapping is an encoder-based approach, it can be easily outfitted with encoding models from other sensory modalities or multiple non-linear encoders within a single modality [26]. However, if there is no retinotopic variation of the encoder feature, then one should not expect to see meaningful variability in the DETI maps. We suspect that is why we did not observe any meaningful spatial variation of orientation biases because the density of neurons tuned to many different orientations does not vary much retinotopically [3,61]. In addition to ‘low-level’ encoders, the DETI procedure can also accommodate ‘higher level’ encoder models derived from human labeling of scenes so long as they vary parametrically. Such an approach would enable the mapping of neural activity to image regions linked to spatially localized task relevant information, thereby providing an opportunity to understand how early transformational states map onto later categorical representations [21], though more complex task designs may require different parameterizations of the DETI map processing pipeline–a possibility that we are currently exploring.

Another useful aspect of DETI mapping is that it can incorporate task-based encoders. We did not include task behavior into the mapping reported here because the task that we employed was only to keep participants engaged and paying attention to the images and was not intended to be used as a behavioral encoder. A proper linking of the DETI maps to behavior would require the use of several tasks because the information (ranging from low to high level) needed to complete those tasks is often correlated, making claims about how and when neural signals ‘inform’ a specific task misleading if there are no other tasks involved [21]. Nevertheless, we conducted an exploratory analysis of the behavioral responses within the DETI mapping paradigm. The task asked participants to assess how cluttered each image is on a 1–4 scale (1 being not cluttered and 4 being very cluttered). We first averaged the clutter scores for each image within participants (i.e., each image is rated 30 times), then across participants. We then mapped the average scores across images to each pixel location by regressing the clutter scores against each encoder’s output. This is the same analysis described in the DETI mapping pipeline for the image-general analysis, only here, clutter scores were used instead of the eigenvectors take from the VEP time windows. The results are reported as supplementary material (**S7 Fig**) in DETI map form, as well as for each encoder’s R^2^ map. The results of that analysis show that low SF encoders seemed to be useful for this task in the lower portion of the images (corresponding to the earlier DETI maps), with higher SFs being mostly scattered but with a tendency to be denser near the central portion of the images (somewhat related to the later DETI maps). However, given that there is just one task in the experiment that was conducted in the current study, caution must be used in interpreting how the encoders link to this task.

As powerful as the DETI mapping procedure can be, it is not without its limitations. Because the procedure maps VEP variance to image variance in encoder space, any covariation between image regions will result in identical tagging, thus complicating any assessment seeking to disambiguate those regions. While sphering the encoder space would help with that problem [21], it may result in new problems. For example, by using scenes, we are presenting observers with the natural covariation in image statistics that are typical of their environment. Decorrelating the covariation would likely create statistical features that are unusual to observers and may produce results may not reflect normal processing. The analyses that we conducted on the DETI maps are not immune to the problem of spatial covariation, so we conducted an analysis of that covariation to explore the extent to which such relationships contributed to those results. Specifically, we examined the extent to which the regional differences in transformational states reflect unique encoder information contained within those regions. To do that, we regressed the encoder responses at corresponding pixel locations between the nine spatial windows used in the image-specific analysis for each image, and then averaged the pixel R^2^s within each window, and then across images. The resulting window-wise R^2^ matrices (averaged over 0.25–0.75 cpd for LSF, and 2.0–8.0 cpd for HSF) are shown in **S8 Fig**. The results of that analysis showed that the spatial covariation was limited to nearby regions and degraded with increased distance over image space. To examine whether this was a specific feature of the images that we used in the current study, we ran the same analysis on all 2500 images in our database and yielded results that were virtually identical to those reported in **S8 Fig**. Another limitation of the DETI procedure is that it gives the best SF tagging distribution when the set of stimuli used to generate the VEP data have a broad range of amplitude spectrum slopes, meaning that the variance across SFs should be quite large. However, if drawing stimuli from a large database of images, an image state-space sampling procedure (see Materials & Methods) will ensure that such a spectral slope distribution is obtained. It’s also worth noting that having a broad range of amplitude spectra slopes within the stimulus set makes the DETI mapping procedure robust to small stimulus sets. Specifically, we found that if that distribution is maintained, the DETI mapping performs well down to about 25 images. Beyond that point, the number of statistically significant pixels drops dramatically.

Limitations aside, the results presented here show that the DETI mapping procedure holds much potential to advance our understanding of the spatiotemporal coding of visual information. One of the most striking results afforded by DETI mapping is the spatiotemporal asymmetry of early scene region SF coding. Such an asymmetry over SF, image space, and time cannot be explained by static image statistics and would not be expected from a simple linear model based on retinotopic mapping of SF preference and suggests a possible prioritization of different image regions as time advances. Further, the results from image-specific local region analysis show that different locations *within* image regions undergo a relatively unique transformational process over time, with the largest difference being between the upper and lower portions of image space. The differential SF transformational states that DETI mapping revealed suggests that the temporal coding of visual information (here indexed by log-Gabor SF power) over image space and time is far more complex than a global coarse-to-fine analysis.

## Materials & methods

### Ethics statement

This study was reviewed and approved by Colgate University’s Institutional Review Board, and all participants gave written informed consent before participating.

### Apparatus

All stimuli were presented on a 23.6” VIEWPixx/EEG scanning LED-backlight LCD monitor with one ms black-to-white pixel response time. Maximum luminance output of the display was 100 cd/m^2^, with a frame rate of 120 Hz and resolution of 1920 x 1080 pixels. Single pixels subtended .0382° of visual angle as viewed from 35 cm. Head position was maintained with an Applied Science Laboratories (ASL) chin rest.

### Participants

A total of 35 participants were recruited for this experiment. Of those, 8 failed to complete both recording sessions and 3 were excluded for having fewer than 50% valid trials following artifact rejection. The age of the remaining 24 participants (13 female, 22 right-handed) ranged from 18–21 (median age = 18). All participants had normal (or corrected to normal) vision as determined by standard ETDRS acuity charts and were compensated for their time.

### Stimuli

Stimuli were selected from a large database of real-world scenes consisting of 2500 photographs that varied in content from purely natural to purely carpentered (both indoor and outdoor), with various mixtures of natural/carpentered environments in between [38]. All images were 512 x 512 pixels and converted to grayscale using the standard weighted sum conversion in MatLab.

For the purposes of stimulus presentation and analysis, all images were calibrated according to the following procedures. First, each image was fit with a hard-edge circular window (with a diameter of 512 pixels) whereby all pixels that fell outside of the circular window were set to zero (i.e., we’re only interested in the 206,643 pixels that were presented to the participants as described later in this section). Next, each image was converted to an array, I(y), that included only the pixels that fell within the circular window and were made to possess the same root mean square (RMS) contrast and mean pixel luminance.

Root mean square contrast is defined as the standard deviation of all pixel luminance values divided by the mean of all pixel luminance values. Image arrays were set to have the same RMS contrast and zero mean using the following operations.

$$I_{sc}=\frac{1}{2}*\frac{I_{zm}}{\max\left|I_{zm}\right|}$$

with I_zm_ defined as:

$$I_{zm}=I(y)-\left(\frac{1}{Y}\sum_{i=1}^{Y}I(y_{i})\right)$$

The pixels values of each image array are first normalized to fall between [-.5 .5] with zero mean as follows,

$${RMS}_{sc}=2*\sqrt{\frac{1}{Y-1}\sum_{i=1}^{Y}{I_{sc}(y_{i})}^{2}}$$

We then calculated an RMS scaling factor, S_rms_ = (2*RMS_t_)/RMS_sc_, with RMS_t_ set to a reasonable target RMS value. By reasonable, we mean a value that did not result in significant (> 5%) clipping of the resulting pixel values. That value was 0.20 for the images used in the current study. Finally, each image array was scaled to have an RMS equal to RMS_t_ and reassign to I(y) as follows: I(y) = 127*(I_sc_*S_rms_). Note that scaling by 127 puts the scaled pixel values of I(y) back in the original range of I_zm_.

Stimulus images were selected according to an image state-space sampling procedure as follows. All images in the database were left in vector form after RMS normalization (described above). When in array form, each cell constitutes a coordinate in a high-dimensional image state-space where each coordinate takes on a pixel luminance value ranging from [–127, 127]. An angular distance matrix was then constructed by calculating the angular distance (in degrees) between each image array and every other image array as follows:

$$\theta =arcos\left(\frac{\overset{⃑}{I_{i}}∙\overset{⃑}{I_{j}}}{\left\|\overset{⃑}{I_{i}}\|*\|\overset{⃑}{I_{j}}\right\|}\right)*\frac{180}{\pi }$$

From there, the angular distance matrix was projected into a lower dimensional space (3D) via t-distributed stochastic neighbor embedding (t-SNE) [62]. Projecting the images into this space enables a general lower dimensional organization of images based on their structural attributes defined by pixel luminance.

Stimuli were selected by uniformly sampling 80 images from the lower dimensional t-SNE space in order to increase the probability of the different regions in our image state-space being represented in the stimulus set. We chose to use 80 images to have as many images as possible while keeping the overall length of the recording sessions to a practical limit. All selected images maintained their RMS of .20 (defined above) but had their mean pixel luminance set to 127 and then fit with a circular linear edge-ramped window (512-pixel diameter, ramped to the mean pixel luminance) to obscure the square frame of the images. That step ensures that the contrast changes at the boundaries of the image were not biased to any particular orientation [63–64].

### Experimental procedure

The experiment consisted of two recording sessions, each ranging between 50–55 min. Within each session, all 80 stimuli were presented 15 times, resulting in a total of 30 presentations per image over both recording sessions (stimulus presentation order was randomized). Each trial began with a 500 ms fixation followed by a variable duration (500–750 ms) blank mean luminance screen to allow any fixation-driven activity to dissipate. The blank screen was immediately followed by the stimulus interval (500 ms) that was then followed by a variable 100–250 ms blank mean luminance screen, followed by a response screen. The response screen prompted the participant to rate the visual clutter of the scene stimuli on a scale of 1–4 (1 being not cluttered and 4 being very cluttered) using a button box (response time was unlimited).

### EEG recording and processing

All Continuous EEGs were recorded in a Faraday chamber using Electrical Geodesics Incorporated’s (MagStim EGI) Geodesic EEG acquisition system (GES 400). All EEGs were obtained by means of Geodesic Hydrocel sensor nets consisting of a dense array of 128 channels (electrolytic sponges). The on-line reference was at the vertex (Cz), and the impedances were maintained below 50 kΩ (EGI amplifiers are high-impedance). All EEG signals were amplified and sampled at 1000 Hz. The digitized EEG waveforms were first highpass filtered at a 0.1 Hz cut-off frequency to remove the DC offset, and then lowpass filtered at a 45 Hz cutoff frequency to eliminate 60 Hz line noise.

Continuous EEGs were divided into 600 ms epochs (99 ms before stimulus onset and 500 ms of stimulus-driven response). Trials that contained eye movements or eye blinks during data epochs were excluded from analysis via magnitude thresholding followed by visual inspection. Additionally, all epochs were subjected to algorithmic artifact rejection whereby voltages exceeding +/- 100 μV or transients greater than +/- 100 μV were omitted from further analysis. These trial rejection routines resulted in a median of 9% (range 3% - 29%) of trials being rejected across participants. Each epoch was then re-referenced offline to the net average, and baseline-corrected to the last 99 ms of the blank interval that preceded the image interval. Finally, VEPs were constructed for each participant by averaging the processed epochs across trials for each image at each electrode, resulting in a 128 x 600 x 80 VEP data matrix for each participant. The full dataset (34Gb) can be downloaded here https://pbsc.colgate.edu/~bchansen/HansenGreeneField2021/HansenGreeneField_Data.zip.

### Encoder model details

Visual evoked potentials constitute the sum of neural activity (post-synaptic potentials) at the circuit level. Given the retinotopic mapping of the visual cortices [34], the VEPs measured on the scalp likely stem from a summation of the underlying responses tuned to different image attributes at different image locations. If the majority of the summation arises from early visual cortical processes [1], then we can expect a good portion of the sum to be explained by contrast in different bands of spatial frequency and orientation. As a first approximation to model the relative response of differently tuned neurons at each location in our stimuli, we used a filter-power encoding model based on log-Gabor filters [38]. Specifically, the model consists of 7 filters, each tuned to a different peak spatial frequency (0.25, 0.50, 0.75, 1, 2, 4, 8 cpd) and all orientations (i.e., a log ‘doughnut’ filter in the Fourier domain). The spatial frequency bandwidths (full width at half height) of the filters scaled with peak spatial frequency such that they were broader at lower spatial frequencies and narrower at higher spatial frequencies: 2.3, 2.3, 2.0, 2.0, 1.75, 1.5, and 1.0 octaves respectively [2–3]. We chose 7 peak encoder frequencies in order to tile as much of the frequency domain as possible within minimal overlap while minimizing filter clipping at the highest frequencies in the Fourier domain.

### Stimulus representation in encoder space

All image filtering was conducted in the Fourier domain using the images in matrix form. To minimize edge effects in the Fourier domain due to the non-periodic nature of scene images, the images were symmetrized prior to taking the Fourier transform. Each symmetrized image was submitted to the 2D discrete fast Fourier transform to obtain H(u,v) as follows:

$$H(u,v)=\frac{1}{XY}\sum_{x=1}^{X}\sum_{y=1}^{Y}I(x,y)e^{-j2\pi (\frac{ux}{X}+\frac{vy}{Y})}$$

where I(x,y) represents a given image, with X and Y representing the dimensions of the symmetrized image. Next, the amplitude spectrum was calculated according to:

$$A(u,v)=\sqrt{{H_{R}(u,v)}^{2}+{H_{I}(u,v)}^{2}}$$

with H_R_(u,v) and H_I_(u,v) representing the real and imaginary parts of H(u,v), respectively. For filtering convenience, the amplitude spectrum, A(u,v) was shifted to polar coordinates and in this form will be denoted as A(*f*,θ), with *f* serving as the index along the radial (i.e., spatial frequency) dimension, and θ as the index along the theta (i.e., orientation) dimension.

Each image’s amplitude spectrum was then multiplied by a 2D log-Gabor filter. Log-Gabor filters in the Fourier domain consist of a log-Gaussian function along the *f* axis and a Gaussian function along the θ axis, which are then combined by multiplying a 2D log-Gaussian filter (i.e., a log ‘doughnut’ filter) with a 2D Gaussian ‘wedge’ filter. The construction of the 2D log-Gaussian filter, L_gaus_(*f*, θ), took place in the same polar coordinate frame as A(*f*,θ). Thus, for each θ axis, L_gaus_(*f*) was modulated as follows.


$$L_{gaus}(f,\theta )=exp\left\{-[\frac{{ln(\frac{f}{f_{peak}})}^{2}}{2*ln(\frac{f_{\sigma }}{f_{peak}})}]\right\}$$

Where *f* increases with spatial frequency (radial distance), *f*_*peak*_ represents the peak of the function, and *f*_*σ*_ represents the SF bandwidth of the filter. Next, a 2D Gaussian function (modulated across θ in radians) about a central orientation was generated as follows.


$$G_{\theta }(f,\theta )=exp\left\{[\frac{-\theta^{2}}{2*\theta_{\sigma }}]\right\}$$

The log-Gabor filter, LG(*f*, θ), was then constructed by multiplying G_θ_(*f*, θ) by L_gaus_(*f*, θ).

The filtered amplitude spectra and corresponding phase spectra were then inverse Fourier transformed back into the spatial domain with the image in its original orientation cropped from the symmetrized version. We then took the natural log of the squared filter responses (i.e., each pixel location across all filters and images was expressed as log power). Representing the filtered images in the spatial domain allowed us access to the encoder responses at each pixel coordinate across all images. The code used to create the encoders and generate the encoder space can be downloaded here https://pbsc.colgate.edu/~bchansen/HansenGreeneField2021/EncoderModel.zip.

### Visualizing the transformational states of scenes over time

To provide a rough visualization of how the images may appear in different transformational states, we ‘reconstructed’ example images using an approach that is similar to the SF bubbles technique in the spatial domain [55] using the participant-averaged VEP data. That process began by first choosing an image and filtering it with the same set of log-Gabors that were used in the DETI mapping procedure. Those filter responses were later mapped to different locations in the reconstruction. Next, we converted each electrode’s fully-tagged image-specific DETI map for a given image for a given time point to a set of seven binary maps, one for each encoder, and then summed those maps (pixel-by-pixel) across all electrodes. That process resulted in 2D histograms for each encoder (similar to what was done for the marginal analysis carried out in the image-general DETI map analysis). Next, for any given pixel coordinate, we selected the encoder that had the highest electrode sum. We then sampled a window centered on the corresponding pixel of the image that had been filtered with the selected encoder. The diameter of that window scaled with encoder peak SF such that lower SFs had larger windows. Specifically, window diameter allowed for 1.5 periods of a given encoder’s SF. That sample was then weighted with a normalized Gaussian (normalized by area) and then summed with the corresponding pixel location in the reconstructed image template. This process was repeated for all pixels.

### Replication data set

EEG data were collected to construct VEPs were recorded from human participants (n = 23) while they viewed 150 scene images sampled from a variety of environments (using the same image state-space sampling procedure described above) as a part of another study [39]. The experiment consisted of one recording session (50–55 min in length). Each of the 150 stimuli were presented 6 times. The presentation sequence was identical to that used in the current study. However, the task required participants to categorize (via mouse click) each image as indoor, urban, or natural. EEG recording and processing were identical to that used in the current study. The final data set was constructed by averaging across participants.

## Supporting information

S1 Fig **A)** All EEG data were collected with Geodesic Hydrocel sensor nets consisting of a dense array of 128 channels. Above is the topographic representation of our sensor nets with the posterior electrodes that we included in our analysis highlighted in red. The posterior electrodes were chosen because VEPs recorded at those sites are known to carry retinotopically selective spatial frequency (SF) information. **B)** Posterior electrode plots showing each encoder’s R2 tuning function averaged over time. Specifically, we averaged across all instances of each encoder’s tag within each electrode’s DETI map at each time point for each participant, and then averaged across all time points and then across participants. All tuning functions have been normalized to the maximum peak within each plot (y-axis). The x-axis shows peak SF for each encoder (enlarged in the lower left corner). The results show largely similar tuning functions at each electrode, thereby justifying the use of selecting the largest R2 to tag each pixel in the DETI maps.
(TIFF)

S2 Fig Replication analyses conducted on a data set that was collected for another study (see Materials & Methods for further detail). Because this is a group analysis (N = 23) where each image is only presented 6 times, this data set used for these analyses basically consists of a single noisy participant with ~168 repetitions. **A)** Example R^2^ maps from two different electrodes and time points. The DETI maps for each example are shown in the upper left of each set of R^2^ maps. Each R^2^ map shows significant R^2^s at each pixel location in image space. The color bar for each map shows R^2^. **B)** Example encoder R^2^ tuning functions for the two DETI maps shown in (A), averaged over all instances of each encoder’s tag in the DETI maps (y-axis is averaged R^2^, x-axis is encoder peak SF). The shaded region of each trace shows the 95% confidence interval over all instances of pixels for each encoder. Given the noise in that data set, the replication analysis results are consistent with those reported in **Figs 4** and **7**.
(TIFF)

S3 Fig Results from the encoder tag probability over time analysis for the orientation tuned encoders. As with the SF probability over time analysis, we calculated the probability of observing pixels tagged with any given encoder’s peak orientation by summing the number of pixels tagged by each encoder for each electrode at each time point and then dividing each sum by the total number of visible pixels in the stimuli. Unlike the SF probability by time analysis, the orientation DETI mapping does not reveal any differences across the ventral-posterior to dorsal-posterior electrodes. However, there is a tendency for the horizontally tuned encoders (90° = horizontal) to be overall less prevalent than the other encoder orientations (note that the y-axes are different across encoder orientation). Please view the accompanying movie for a complete depiction of how different orientation DETI maps evolve over time https://pbsc.colgate.edu/~bchansen/HansenGreeneField2021/HansenGreeneField_SupplFigure3_Movie.mp4.
(TIFF)

S4 Fig Marginal analyses of the 2D R2 encoder maps. The row-wise marginal analysis (top) consists of an average across the R^2^ maps from left to right for each time point. The columns of that plot were first normalized over time and then normalized again within each column to emphasize encoder fit magnitude over time and space. The column-wise marginal analysis was carried out the same way, but from top to bottom of the R^2^ maps (the normalization therefore took place row-wise).
(TIFF)

S5 Fig Encoder R2s over time, averaged across participants. The y-axes show participant-averaged R^2^s, with time (ms) on the x-axes. Each trace is from a specific electrode. The electrode traces are color-coded topographically in two ways (illustrated at the top of each set of plots). The left-hand plots are coded from ventral-posterior to dorsal-posterior portions of the scalp, with the right-hand side coded from left to right across the scalp.
(TIFF)

S6 Fig Image-specific DETI mapping procedure results using the Benjamini-Hochberg correction procedure with a false discovery rate of 5%. The axes of the results presented above is identical to that shown in **Figs 10–13**.
(TIFF)

S7 Fig Results from incorporating the behavioral results (clutter scores for each image) into the DETI analysis. The left-hand side shows the participant averaged R^2^s over time for each electrode (color coded from posterior ventral to dorsal scalp). The right-hand side shows the behavioral R^2^ maps along with marginal means (windowed regions excluded from the marginal mean) (see the main article for details). The behavioral DETI map is shown in the upper left of the set of R^2^ maps. Each R^2^ map shows significant R^2^s at each pixel location in image space. The color bar for each map shows R^2^.
(TIFF)

S8 Fig R2 matrices showing the relationship between the encoder responses at each pixel coordinate within one of the nine image regions and the corresponding pixel coordinate in every other patch region. R^2^s are averaged across all pixel coordinates within each patch, and then averaged across the lower SFs (LSF; left hand matrix) and higher SFs (HSF; right hand matrix). The color bar shows R^2^.
(TIFF)
