# Supplementary figures and images for: Dynamic Electrode-to-Image (DETI) mapping reveals the human brain’s spatiotemporal code of visual information
Source: PLoS Comput Biol. 2021 Sep 27;17(9):e1009456. doi: 10.1371/journal.pcbi.1009456 (PMC8496831; doi:10.1371/journal.pcbi.1009456)

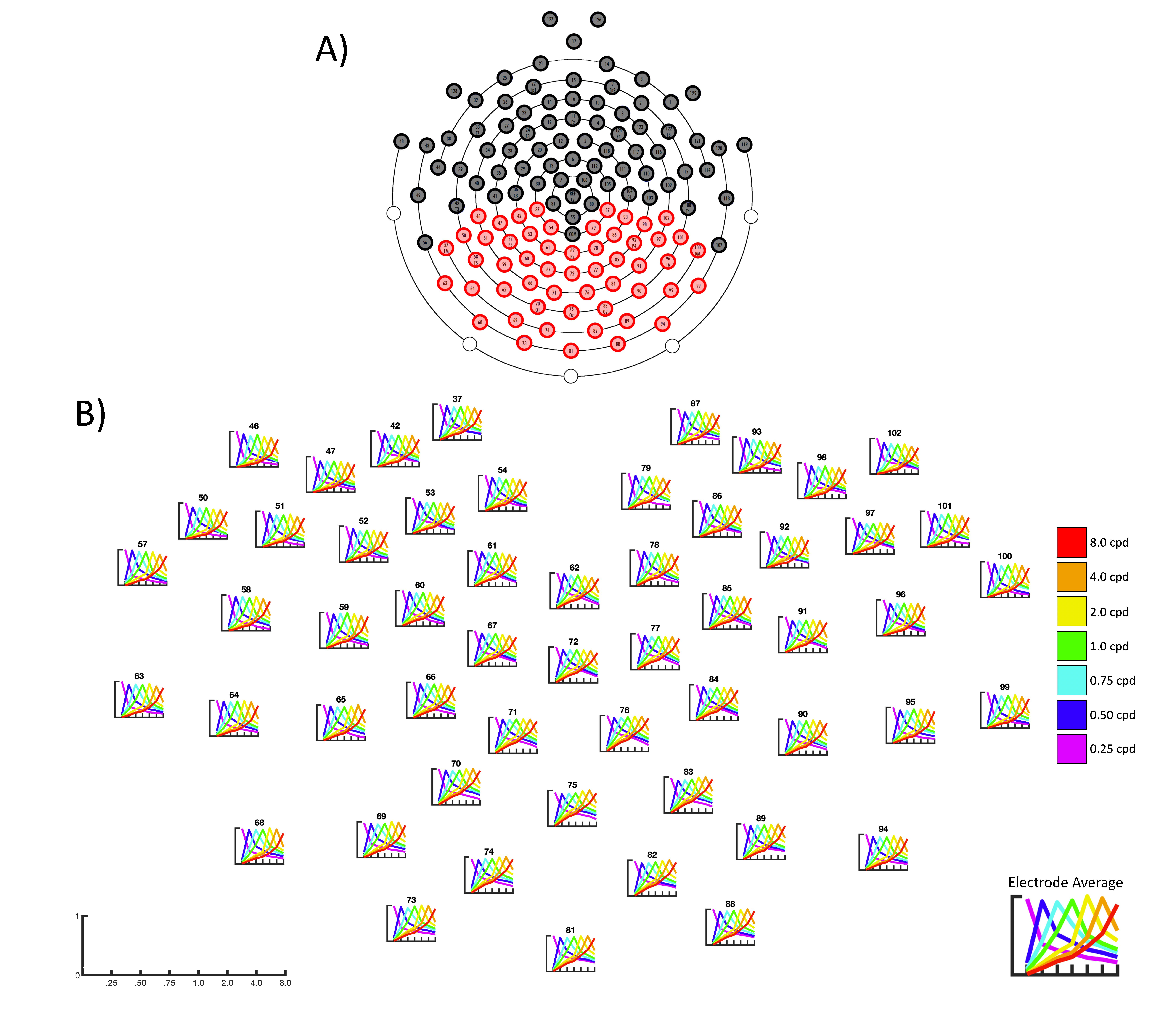

Supplement: S1 Fig — A) All EEG data were collected with Geodesic Hydrocel sensor nets consisting of a dense array of 128 channels. Above is the topographic representation of our sensor nets with the posterior electrodes that we included in our analysis highlighted in red. The posterior electrodes were chosen because VEPs recorded at those sites are known to carry retinotopically selective spatial frequency (SF) information. B) Posterior electrode plots showing each encoder’s R2 tuning function averaged over time. Specifically, we averaged across all instances of each encoder’s tag within each electrode’s DETI map at each time point for each participant, and then averaged across all time points and then across participants. All tuning functions have been normalized to the maximum peak within each plot (y-axis). The x-axis shows peak SF for each encoder (enlarged in the lower left corner). The results show largely similar tuning functions at each electrode, thereby justifying the use of selecting the largest R2 to tag each pixel in the DETI maps. (TIFF) [file pcbi.1009456.s001.tiff]

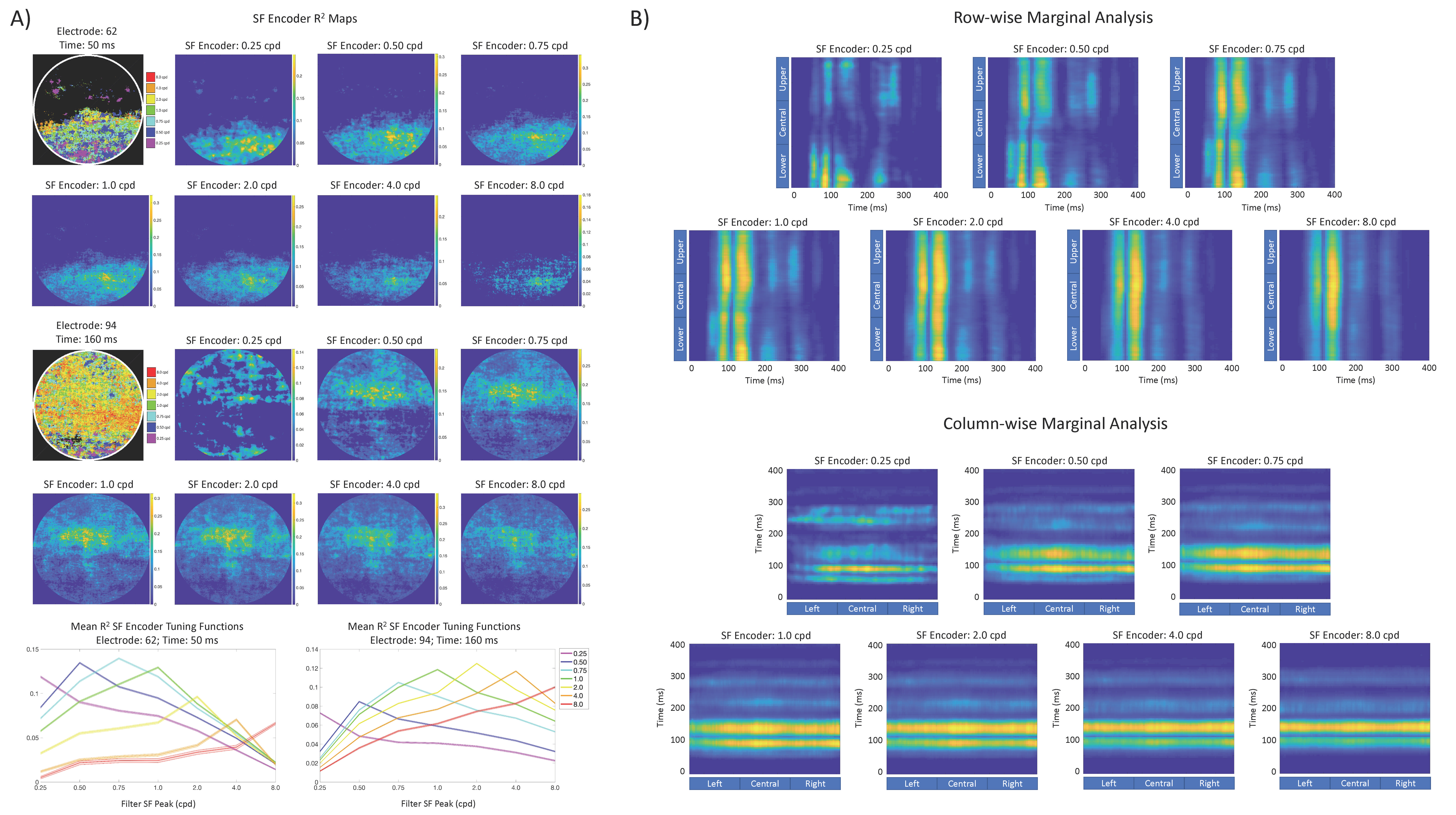

Supplement: S2 Fig — Because this is a group analysis (N = 23) where each image is only presented 6 times, this data set used for these analyses basically consists of a single noisy participant with ~168 repetitions. A) Example R2 maps from two different electrodes and time points. The DETI maps for each example are shown in the upper left of each set of R2 maps. Each R2 map shows significant R2s at each pixel location in image space. The color bar for each map shows R2. B) Example encoder R2 tuning functions for the two DETI maps shown in (A), averaged over all instances of each encoder’s tag in the DETI maps (y-axis is averaged R2, x-axis is encoder peak SF). The shaded region of each trace shows the 95% confidence interval over all instances of pixels for each encoder. Given the noise in that data set, the replication analysis results are consistent with those reported in Figs 4 and 7. (TIFF) [file pcbi.1009456.s002.tiff]

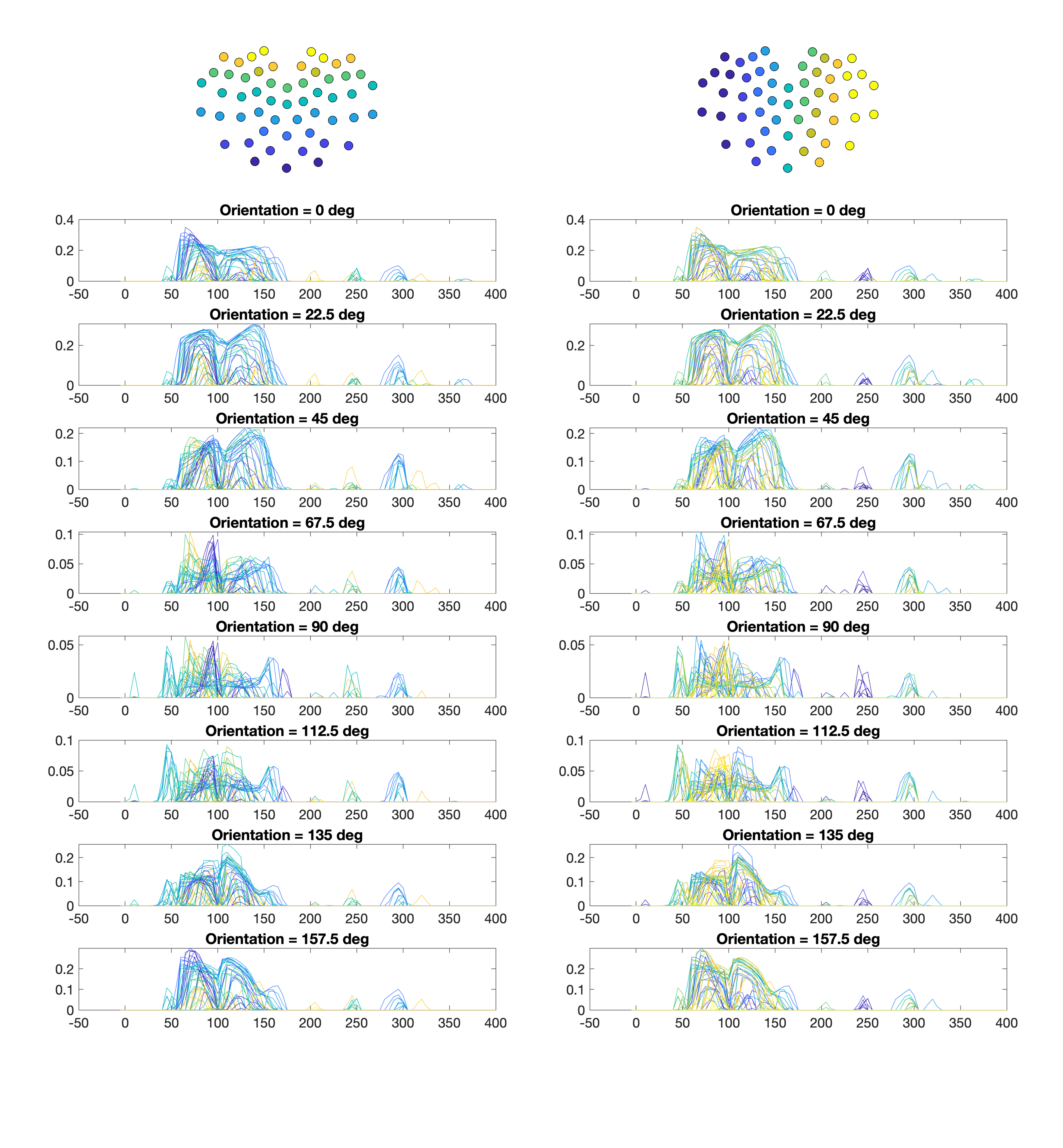

Supplement: S3 Fig — As with the SF probability over time analysis, we calculated the probability of observing pixels tagged with any given encoder’s peak orientation by summing the number of pixels tagged by each encoder for each electrode at each time point and then dividing each sum by the total number of visible pixels in the stimuli. Unlike the SF probability by time analysis, the orientation DETI mapping does not reveal any differences across the ventral-posterior to dorsal-posterior electrodes. However, there is a tendency for the horizontally tuned encoders (90° = horizontal) to be overall less prevalent than the other encoder orientations (note that the y-axes are different across encoder orientation). Please view the accompanying movie for a complete depiction of how different orientation DETI maps evolve over time https://pbsc.colgate.edu/~bchansen/HansenGreeneField2021/HansenGreeneField_SupplFigure3_Movie.mp4. (TIFF) [file pcbi.1009456.s003.tiff]

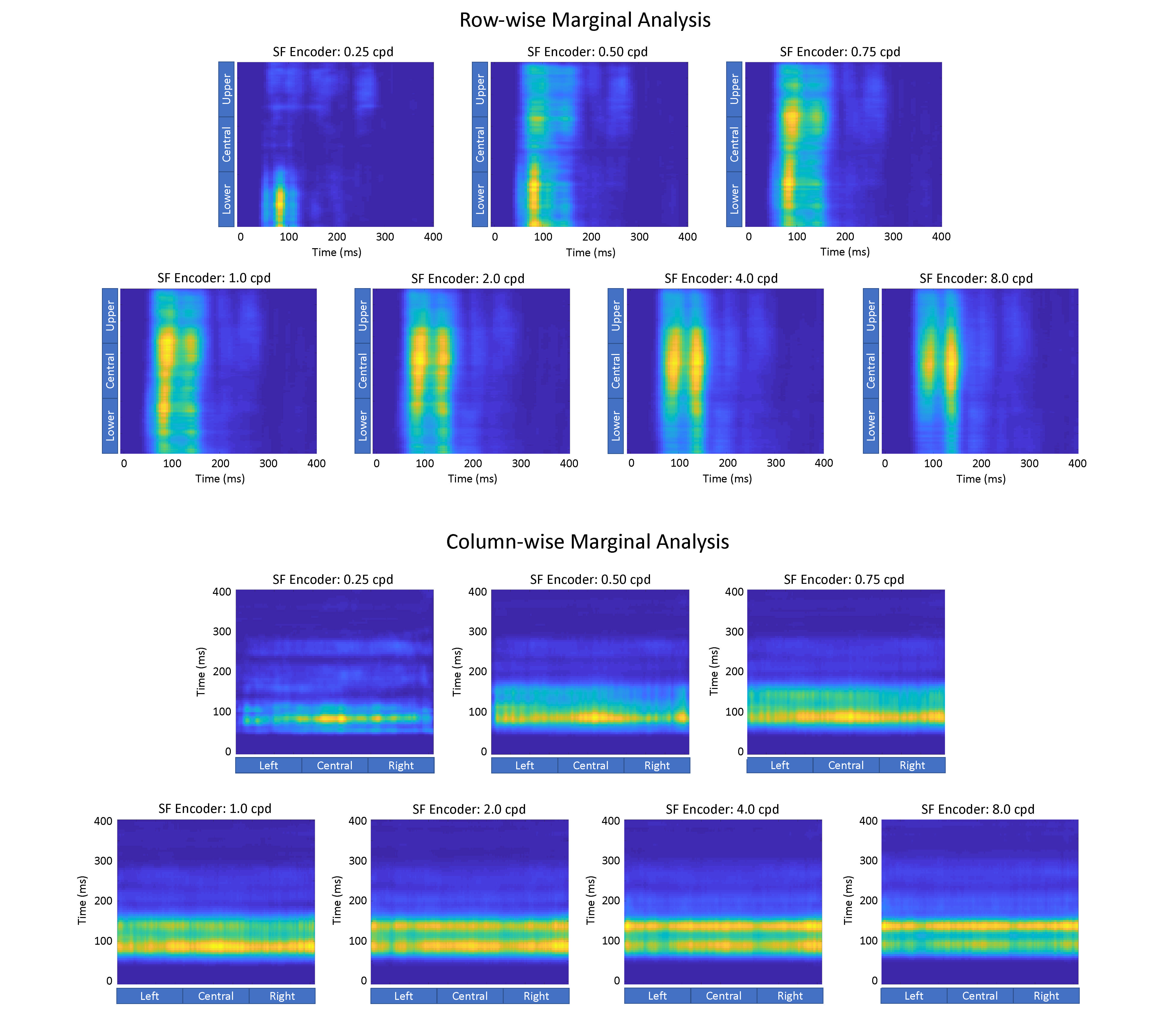

Supplement: S4 Fig — The row-wise marginal analysis (top) consists of an average across the R2 maps from left to right for each time point. The columns of that plot were first normalized over time and then normalized again within each column to emphasize encoder fit magnitude over time and space. The column-wise marginal analysis was carried out the same way, but from top to bottom of the R2 maps (the normalization therefore took place row-wise). (TIFF) [file pcbi.1009456.s004.tiff]

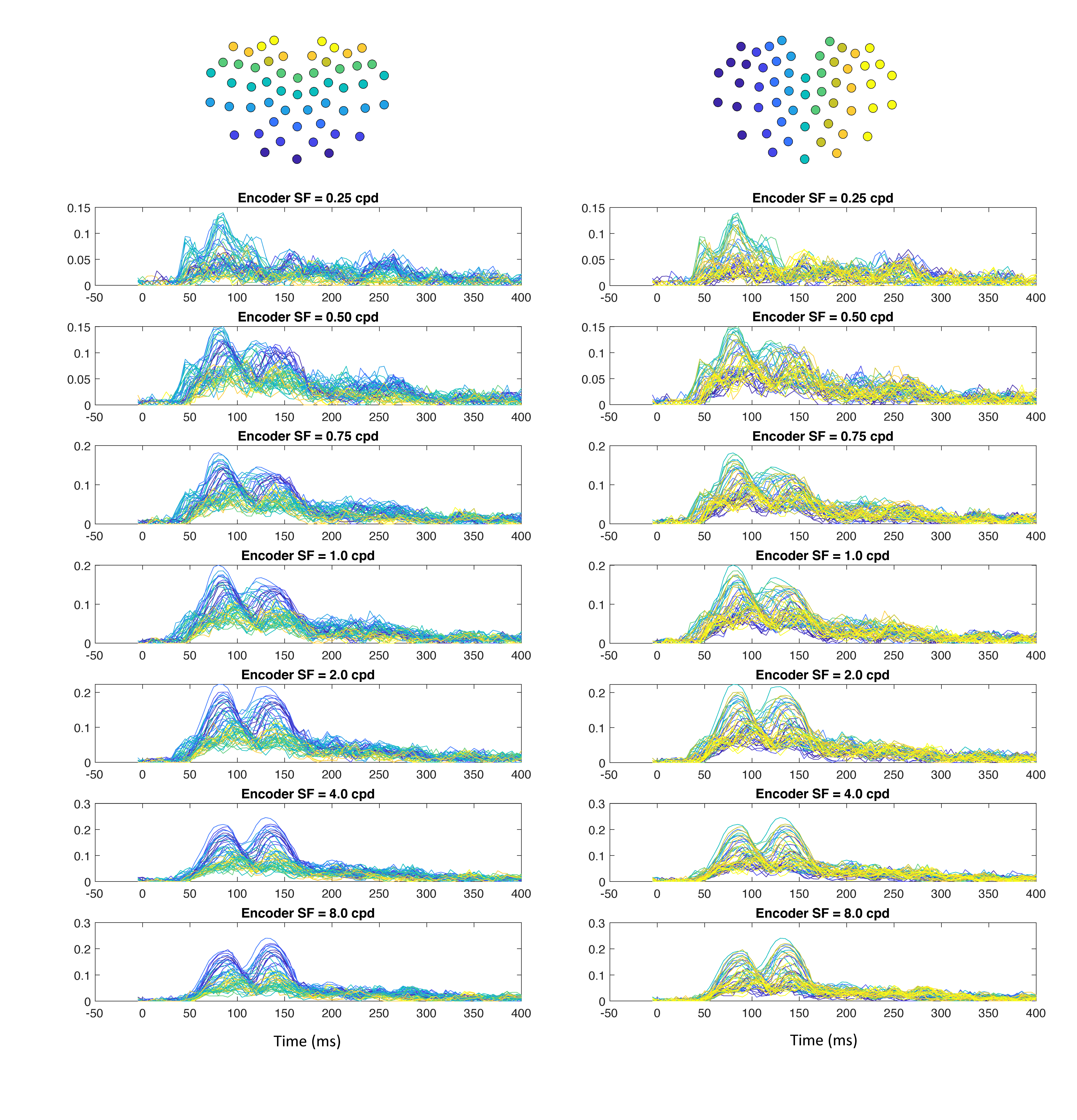

Supplement: S5 Fig — The y-axes show participant-averaged R2s, with time (ms) on the x-axes. Each trace is from a specific electrode. The electrode traces are color-coded topographically in two ways (illustrated at the top of each set of plots). The left-hand plots are coded from ventral-posterior to dorsal-posterior portions of the scalp, with the right-hand side coded from left to right across the scalp. (TIFF) [file pcbi.1009456.s005.tiff]

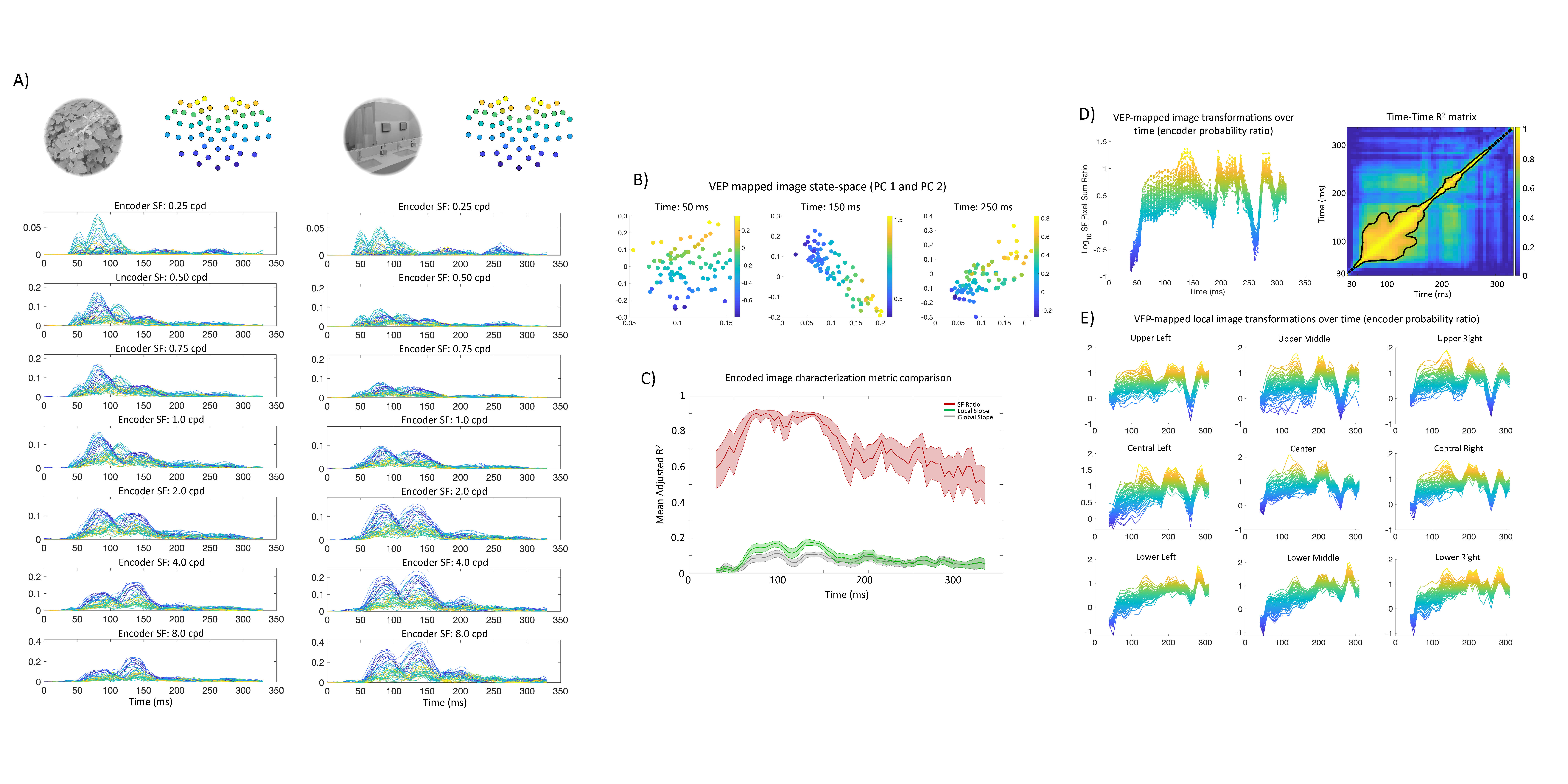

Supplement: S6 Fig — The axes of the results presented above is identical to that shown in Figs 10–13. (TIFF) [file pcbi.1009456.s006.tiff]

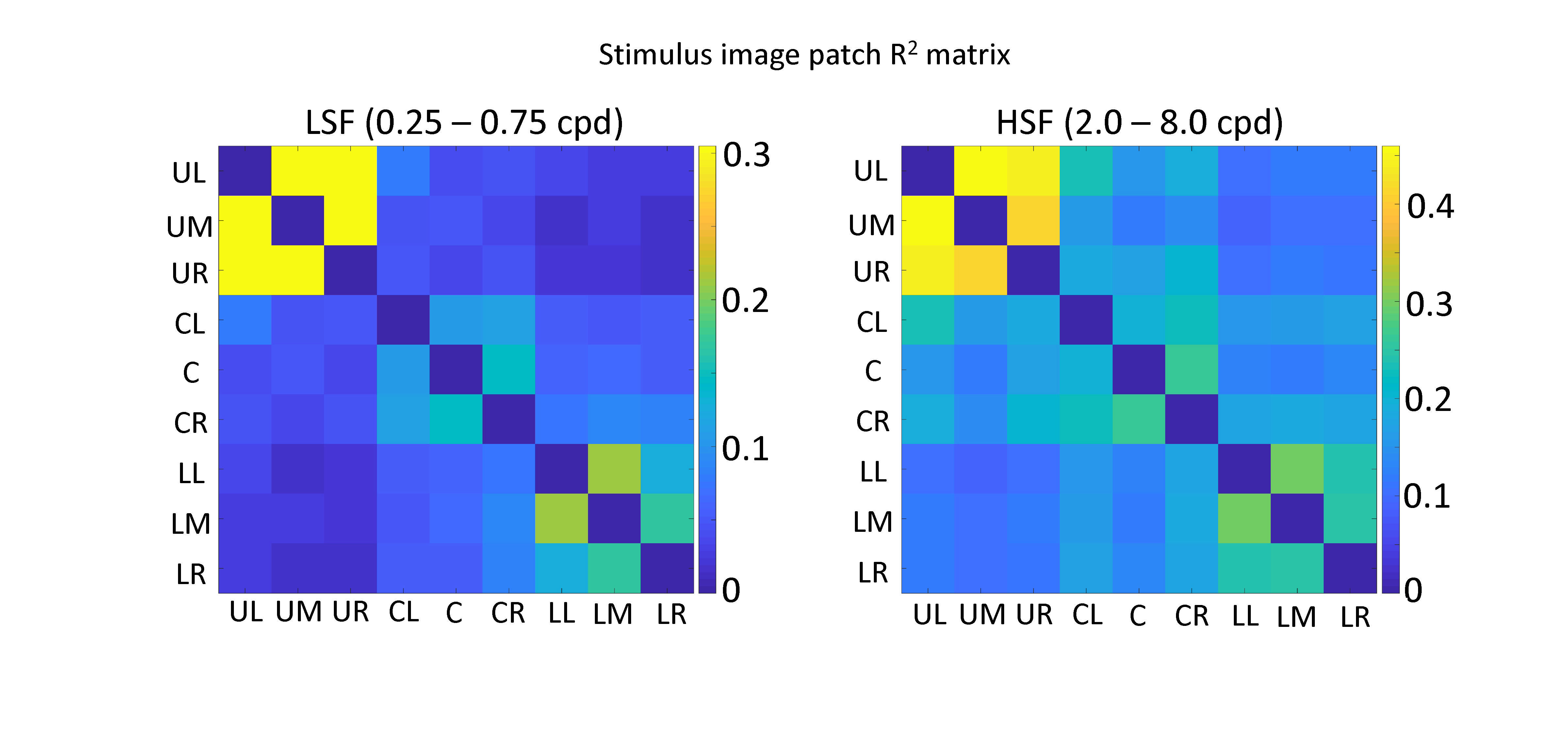

Supplement: S8 Fig — R2s are averaged across all pixel coordinates within each patch, and then averaged across the lower SFs (LSF; left hand matrix) and higher SFs (HSF; right hand matrix). The color bar shows R2. (TIFF) [file pcbi.1009456.s008.tiff]
